# The Burden of Respiratory Syncytial Virus in Children With Acute Otitis Media: A Systematic Review and Meta‐Analysis

**DOI:** 10.1111/irv.70223

**Published:** 2026-02-04

**Authors:** Sebastien Kenmoe, Marshall Dozier, Jingyi Liang, Ruth Jenkins, Harish Nair

**Affiliations:** ^1^ Centre for Global Health, Usher Institute University of Edinburgh Edinburgh UK; ^2^ Library & University Collections University of Edinburgh Edinburgh UK

**Keywords:** acute otitis media, disease burden, respiratory syncytial virus

## Abstract

**Background:**

Acute otitis media (AOM) affects over 709 million individuals globally each year, more than half of whom are children < 5 years. Respiratory syncytial virus (RSV) is a leading viral cause of pediatric respiratory illness. We aimed to estimate the burden of RSV in children < 5 years with AOM

**Methods:**

We performed a systematic review of studies reporting RSV identified through laboratory testing in children < 5 years with AOM. We searched eight databases from January 1, 1996, to May 9, 2025. We extracted data on RSV proportion in AOM and on co‐detection of bacterial pathogens. We reported pooled proportions using random‐effects meta‐analysis.

**Results:**

We included 27 studies encompassing 8342 children with AOM. The pooled proportion of RSV in children with AOM was 16.9% (95% CI 11.0–23.8, *I* ^2^ = 94.9%). RSV proportion was higher in inpatient‐based studies, in studies conducted during peak RSV seasons, and in children aged < 12 months. Among RSV‐positive AOM cases, an estimated 67.4% (95% CI 15.4–100) had at least one bacterial co‐detection. The most frequent bacteria co‐detected were *Streptococcus pneumoniae* , followed by *Haemophilus influenzae* and *Moraxella catarrhalis* .

**Conclusion:**

RSV is a common contributor to AOM in children < 5 years. Our findings indicate that RSV‐associated AOM often involves concurrent bacterial detection. These results highlight the potential impact of recently introduced passive RSV immunization, such as maternal immunization and long‐acting monoclonal antibody, in reducing AOM incidence and its complications. Preventing RSV in early childhood could lower the overall burden of AOM and decrease the need for antibiotics.

## 1 Background

Globally, acute otitis media (AOM) affects over 709 million individuals annually, more than half of whom are children < 5 years [1]. AOM causes ear pain and potential hearing loss with recurrent episodes, drives pediatric consultations and antibiotic use, and imposes substantial healthcare costs [2]. AOM is traditionally viewed as a bacterial illness, but viral respiratory infections play a critical role in its development. Respiratory syncytial virus (RSV) is a major cause of respiratory infections in children < 5 years, responsible for an estimated 33 million lower respiratory tract infections (LRTI) annually, with nearly all children infected by age 2 and many requiring hospital admission [3]. These figures underscore the importance of understanding and mitigating AOM, particularly episodes associated with common viral agents like RSV. Respiratory viruses contribute to AOM by disrupting Eustachian tube function, with RSV carrying the highest risk [4]. RSV modulates both innate and adaptive immunity, altering neutrophil and epithelial cell function, impairing mucociliary clearance, and enhancing local inflammatory mediator release, which can facilitate AOM even in the absence of bacteria. Moreover, RSV infection increases bacterial colonization and enhances bacterial adherence to respiratory epithelium, thereby promoting synergistic virus‐bacteria interactions that contribute to disease. Bacterial co‐detection increases disease severity and complicates treatment [5]. Studies have focused on the role of RSV in AOM, but the overall burden remains unclear. With the recent introduction of RSV immunization programs and other interventions, a comprehensive synthesis of existing evidence is needed to establish a robust baseline before new interventions alter epidemiological patterns. A systematic review of all published studies to date is therefore justified to integrate findings from a field with limited and heterogeneous data, strengthen precision through pooled estimates, and identify persisting evidence gaps that warrant future research. This systematic review aimed to quantify the proportion of RSV in children < 5 years with AOM. We assessed the frequency of bacterial co‐detections in RSV‐associated AOM cases. We examined how age, clinical setting, diagnostic method, and region influence these estimates.

## 2 Methods

### 2.1 Search Strategy

We conducted this systematic review following PRISMA 2020 guidelines [6]. We registered the protocol in the PROSPERO database (CRD42024545528). We searched eight electronic databases: Embase, Scopus, Web of Science, MEDLINE, Global Health, and three Chinese databases (China National Knowledge Infrastructure, Wanfang Data, and Chongqing VIP). We included studies published from January 1, 1996, to May 9, 2025. We used keyword combinations related to “RSV,” “AOM,” and “children.” We adapted the search syntax for each database, including the use of Chinese keywords for Chinese language databases (Table S1). We applied no language restrictions. We manually screened the reference lists of relevant articles to identify additional studies.

### 2.2 Selection Criteria

We included prospective or retrospective observational studies of any design (cohort, cross‐sectional, or case–control) that reported RSV detection in children < 5 years with AOM. We considered studies conducted in inpatients (for those who acquired the condition in the community and were hospitalized with it) or community settings. We included studies that used any RSV diagnostic method (e.g., PCR, viral culture, or antigen testing) in middle ear fluid or respiratory samples. We considered study‐specific definitions of AOM that included common clinical symptoms such as fever, irritability, or otalgia, reported within 7 days of onset. Studies were required to confirm AOM clinically (e.g., by otoscopy), and we considered all AOM definitions (e.g., recurrent AOM or OM with effusion) if RSV testing was reported. We excluded studies conducted exclusively in high‐risk children that did not report separate data for children < 5 years, studies that lacked data on RSV infection, reviews, conference abstracts without complete data, and duplicate publications.

### 2.3 Screening and Data Extraction

Two reviewers (S.K. and J.L.) independently screened the titles and abstracts of identified records. They independently assessed full‐text articles against the eligibility criteria. We documented reasons for exclusion at the full‐text stage and summarized these in a PRISMA flow diagram. We developed a standardized data extraction form to collect information from each included study. Two reviewers (S.K. and J.L.) independently extracted the data. We resolved discrepancies by discussion or by consultation with a third reviewer (H.N.) when necessary. For each study, we recorded publication details (year and country), study design (e.g., prospective cohort and retrospective cross‐sectional), setting (community, outpatient, and hospital), sample size, age range of children, and recruitment period. We collected clinical data including radiological findings, antibiotic and vaccination status (e.g., pneumococcal conjugate vaccine [PCV] and Hib vaccine), case definition of AOM, and details on sequelae and complications. We also collected data on prior antibiotic use and the number of days of parental and child absenteeism. We extracted the number of children with AOM tested for RSV and the number who tested positive. We extracted the number of children with AOM and RSV tested for bacterial co‐detection and the number who tested positive for at least one bacterial pathogen or for specific bacteria. We recorded the RSV detection method (PCR, culture, rapid antigen test, etc.) and the sample type (middle ear fluid, nasopharyngeal swab, etc.) used for RSV or bacterial testing.

### 2.4 Risk of Bias Assessment

We developed a customized checklist based on established quality criteria for observational studies [7]. We assessed key domains including population representativeness, adequacy of the AOM case definition, accuracy of RSV diagnosis, participant characteristics, study duration, reporting of antibiotic status, and statistical methods (Table S2). We classified studies with scores below 4 (out of a maximum of 10) as having high risk of bias, scores between 4 and 6 as moderate risk, and scores above 6 as low risk of bias.

### 2.5 Data Analysis

We summarized the characteristics of included studies qualitatively and in tables. We performed meta‐analyses to estimate the pooled proportion of RSV in AOM cases, with 95% confidence intervals (CIs). We used random‐effects models with proportions transformed using the Freeman–Tukey double arcsine method and Clopper–Pearson CIs [8, 9]. We used the restricted maximum likelihood estimator for between‐study variance [10]. Pooled estimates were obtained using inverse‐variance weighting under the random‐effects model. In studies reporting bacterial co‐detections in RSV‐positive AOM cases, we pooled the proportion of any bacterial (one or more) co‐detection and of specific bacterial species (such as *Haemophilus influenzae* , *Streptococcus pneumoniae* , and *Moraxella catarrhalis* ), with 95% CIs. For study designs such as case–control studies, where AOM was the exposure in RSV‐positive individuals, we reported only the proportion of bacterial co‐detection within the RSV‐positive AOM group. We quantified statistical heterogeneity using the *I* ^2^ statistic (*I* ^2^ > 50% indicating high heterogeneity) [11]. We explored potential sources of heterogeneity through subgroup analyses stratified by setting, country, data collection period (seasonal vs. year‐round), age group, diagnostic sample, and diagnostic method. We performed sensitivity analyses including only studies with low risk of bias and those with no prior antibiotic use before sample collection, because antibiotics could suppress bacterial detection. We assessed publication bias using Egger's test, with a significance threshold of *p* < 0.05 [12]. We conducted all analyses using R software Version 4.4.1.

## 3 Results

**FIGURE 1:**
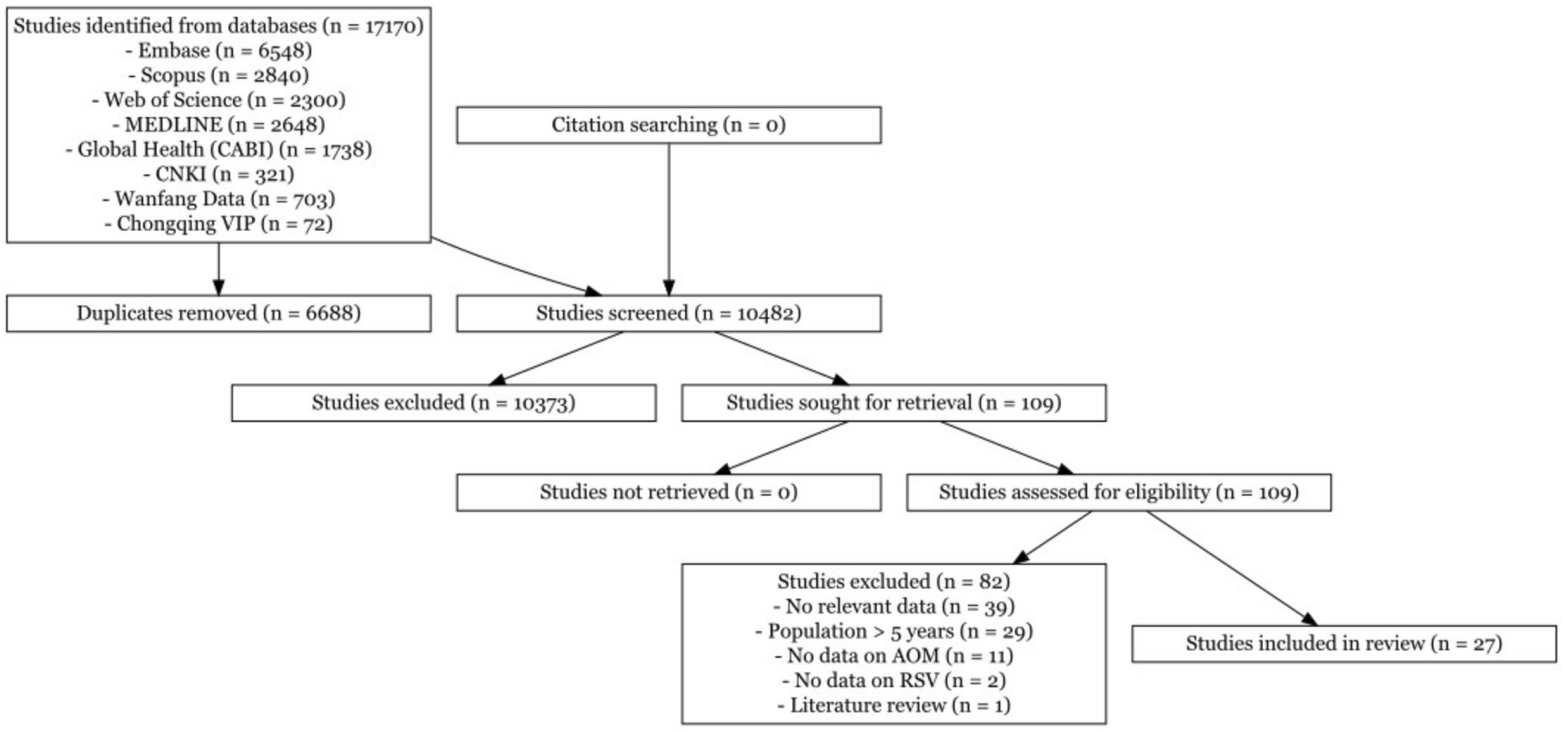
PRISMA flow diagram of study selection.

We identified 17,170 studies from various databases, of which 109 potentially eligible full‐text articles were assessed and 27 studies met the inclusion criteria for this systematic review (Figure 1) [13, 14, 15, 16, 17, 18, 19, 20, 21, 22, 23, 24, 25, 26, 27, 28, 29, 30, 31, 32, 33, 34, 35, 36, 37, 38, 39].

The majority of included studies were cohort studies (66.7%) with prospective data collection (74.1%) (Table S3). Of the included studies, 10/27 (37.0%) used descriptive statistics (counts, percentages, and means), 10/27 (37.0%) employed comparative tests (chi‐square, Fisher, and Mann–Whitney), and 7/27 (26.0%) applied regression models (logistic, generalized estimating equations) with adjustments for covariates such as age, ethnicity, and environmental exposures. Most studies were conducted in urban areas (92.6%) and outpatient settings (51.9%). High‐income countries accounted for most studies (88.9%), with the largest proportion conducted in Europe (59.3%), particularly Finland (33.3%), followed by the United States (14.8%) and Japan (11.1%) (Figure S1). The studies were published between 1997 and 2021, with study durations ranging from 6 to 72 months and data collection mostly performed year‐round (88.9%) between 1993 and 2020. Most participants were < 2 years (59.2%). None of the 27 studies specified chest x‐ray findings, and parental workplace and child absenteeism were not reported. Sequelae details were largely unreported, with only one study (3.7%) mentioning persistent middle ear effusion, early recurrence, and recurrent AOM [25]. Antibiotic use before sample collection was excluded in 29.6% of studies, whereas antibiotic administration was reported in 25.93% of studies, with proportion ranging from 26.4% to 100%. Amoxicillin was the most commonly administered antibiotic, and one study reported higher antibiotic use in RSV‐negative children (*p* = 0.001) [22]. Vaccination status was generally unspecified in the majority of the studies (74.1%), but where available, data indicated various vaccination schedules, including at least one dose of HiB (100% coverage, two studies) and between one and three doses of PCV, with coverage ranging from 2% to 100% across seven studies. In one study, PCV did not significantly influence the proportion of *S. pneumoniae* (vaccinated: 10.0%; unvaccinated: 22.3%; *p* = 0.08) or *H. influenzae* (vaccinated: 35.0%; unvaccinated: 30.0%; *p* = 0.53) [23]. Identified *S. pneumoniae* serotypes in vaccinated children included 14, 18C, 21, and 6A, whereas serotypes 1, 6B, 14, 15B, 15C, 16, 19A, 19F, 21, 23F, 3, 4, 5, 6A, 6B, 9N, and 9V were detected in unvaccinated children. No significant difference was observed in PCV7 serotypes between vaccinated (5.0%) and unvaccinated children (12.7%; *p* = 0.3). Among 80 *H. influenzae* ‐positive episodes, 65 occurred in unvaccinated children and 14 in vaccinated children, all being NTHi strains. AOM diagnosis was most often performed by physicians (29.6%), with diagnostic techniques primarily involving otoscopy (25.9%) or otoscopy with tympanometry (18.5%). The duration of AOM was not specified in most studies (88.9%). Diagnostic specimens for RSV detection were mainly nasopharyngeal samples (33.3%) and middle ear fluid (18.5%). RSV diagnostic tests varied widely, with PCR (18.5%) being the most commonly used. For bacterial detection, 63.0% of studies did not perform bacterial testing, whereas 25.9% used culture methods. Among the 27 studies, 85.2% were considered to have a low risk of bias (Table S4).

### 3.1 Proportion of RSV Infection in Children With Acute Otitis Media

**FIGURE 2:**
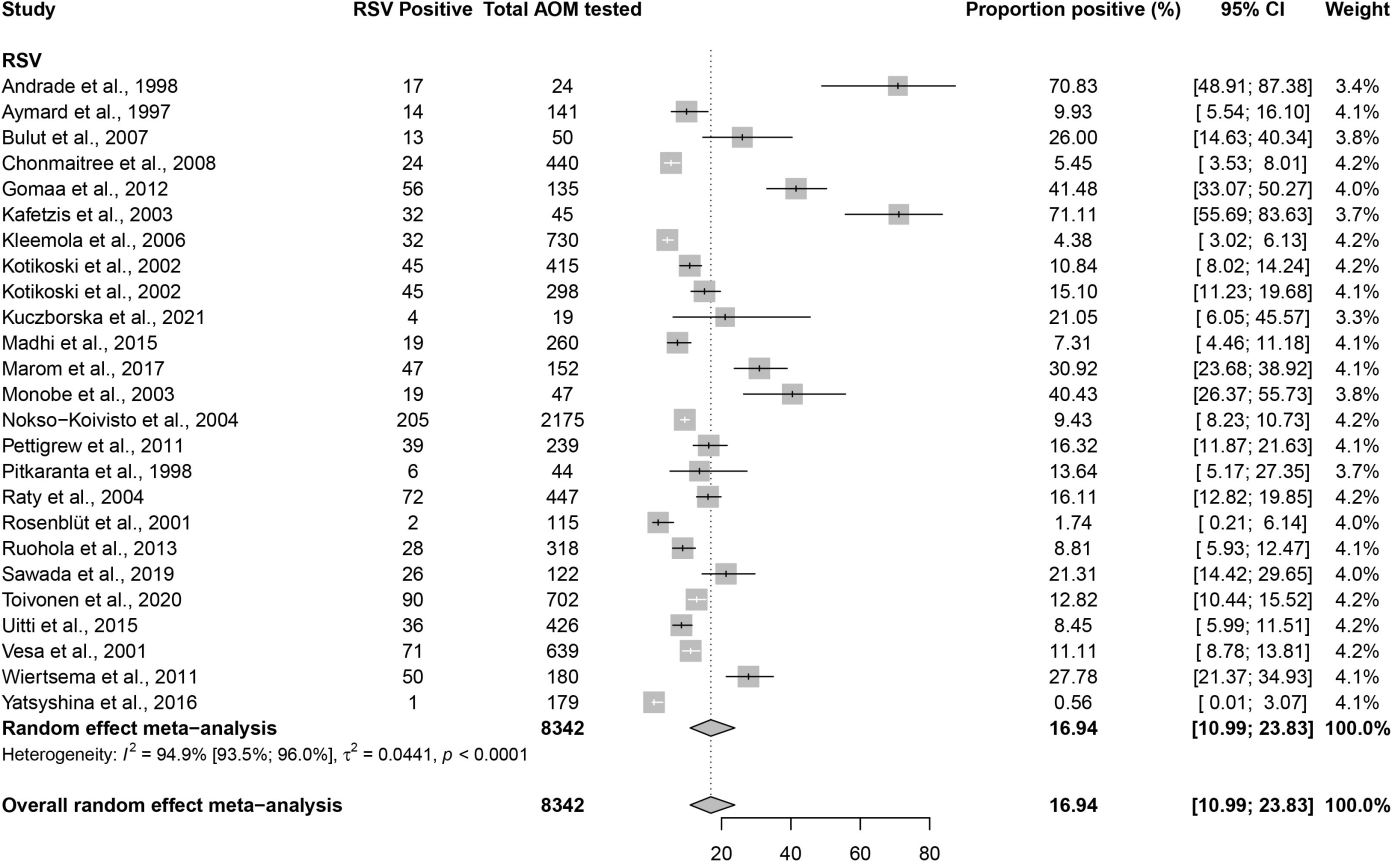
Forest plot showing the proportion of RSV‐positive cases among children with acute otitis media in included studies. Confidence intervals are shown both on the transformed scale used for pooling (thin black lines) and on the back‐transformed proportion scale (thick white lines). The white line may overlap the black line when both intervals coincide.

The pooled overall proportion of RSV among children with AOM across 25 data points was 16.9% (95% CI, 11.0–23.9), with high heterogeneity (*I* ^2^ = 94.9%, 95% CI 93.5–96) (Figure 2). Among studies with a low risk of bias (22 data points), the pooled proportion was slightly lower at 15.4% (95% CI, 9.9–21.8), with similarly high heterogeneity (*I* ^2^ = 94.5%, 95% CI 92.8–95.8). Egger's test suggested potential publication bias for both overall studies (*p* = 0.008) and studies with low risk of bias (*p* = 0.025).

Subgroup analysis shows that RSV proportion among children with AOM was highest in inpatient‐based studies (26.4%; 95% CI, 10.2–46.7), seasonal studies (50.7%; 95% CI, 31.5–69.8), younger children aged 0–11 months (30.9%; 95% CI, 23.8–38.5), and middle ear fluid samples combined with nasal samples (70.8%; 95% CI, 50.8–87.6) (Table S5). The categories within regional, economic, and diagnostic methods subgroups were heterogeneous and represented by a small number of studies, preventing meaningful conclusions.

### 3.2 Proportion of Overall and Etiology‐Specific Bacterial Co‐Detections in Children With RSV‐Associated AOM

The meta‐analysis shows that bacterial co‐detections are common among children with RSV‐associated AOM, with an overall proportion of 67.4% (95% CI, 15.4–100) based on three data points (Table 1). *S. pneumoniae* had the highest proportion at 28.5% (95% CI, 12.8–47.2; 8 data points), followed by *H. influenzae* (24.9%; 95% CI, 5.8–50.6; 6 data points) and *M. catarrhalis* (18.9%; 95% CI, 5.8–36.5; 8 data points). Lower proportions were observed for *Staphylococcus aureus* (4.3%; 95% CI, 0.0–15.6), *Streptococcus pyogenes* (0.3%; 95% CI, 0.0–3.4), and other bacterial pathogens such as *Chlamydophila pneumoniae* , *Mycoplasma pneumoniae* , *Legionella pneumophila* , *Enterococcus faecalis* , and *Escherichia coli* , all with no detected cases. High heterogeneity was present across most bacterial groups, particularly for *S. pneumoniae* (*I* ^2^ = 92%) and *H. influenzae* (*I* ^2^ = 93.1%). Sensitivity analysis of studies without prior antibiotic administration generally showed lower bacterial proportions. Studies considered to be at low risk of bias showed similar bacterial proportion trends. No significant publication bias was detected for any bacteria based on Egger's test results.

**TABLE 1:** Sensitivity analysis of RSV proportion and bacterial codetections (overall and etiology‐specific) in children < 5 years with AOM.

	Prevalence [95% CI]	*N* cases	*I* ^2^ ^a^ (95% CI)	*p* Egger's test
RSV in AOM				
Overall (25 data points)	16.9 [11–23.8]	8342	94.9 [93.5–96]	0.008
Low risk of bias (22 data points)	15.4 [9.9–21.8]	7709	94.5 [92.8–95.8]	0.025
Bacteria 1+ in RSV‐associated AOM				
Overall (3 data points)	67.4 [15.4–100]	176	98.3 [96.9–99.1]	0.094
Antibiotic_before_sampling (2 data points)	51.6 [0–100]	123	98.7 [97.3–99.4]	NA
Low risk of bias (2 data points)	51.6 [0–100]	123	98.7 [97.3–99.4]	NA
NTHi in RSV‐associated AOM^b^				
Overall (2 data points)	56.3 [46.5–65.9]	103	0	NA
Low risk of bias (1 data point)	58 [44–71.4]	50	NA	NA
*Streptococcus pneumoniae* in RSV‐associated AOM				
Overall (8 data points)	28.5 [12.8–47.2]	312	92 [86.6–95.2]	0.978
Antibiotic_before_sampling (3 data points)	17.6 [10.3–26.2]	99	0 [0–89.6]	0.587
Low risk of bias (7 data points)	24.3 [9.3–43.2]	259	90.8 [83.5–94.8]	0.8
*Haemophilus influenzae* in RSV‐associated AOM				
Overall (6 data points)	24.9 [5.8–50.6]	209	93.1 [87.8–96.2]	0.521
Antibiotic_before_sampling (3 data points)	12.4 [3.5–24.6]	99	42.6 [0–82.7]	0.61
Low risk of bias (6 data points)	24.9 [5.8–50.6]	209	93.1 [87.8–96.2]	0.521
*Moraxella catarrhalis* in RSV‐associated AOM				
Overall (8 data points)	18.9 [5.8–36.5]	312	91.4 [85.5–94.9]	0.303
Antibiotic_before_sampling (3 data points)	9 [2.8–17.7]	99	28.1 [0–92.5]	0.411
Low risk of bias (7 data points)	13.9 [3.9–27.8]	259	85.7 [72.4–92.5]	0.403
*Staphylococcus aureus* in RSV‐associated AOM				
Overall (4 data points)	4.3 [0–15.6]	124	70.8 [16.5–89.8]	0.866
Antibiotic_before_sampling (2 data points)	7.7 [0–38.2]	32	75.6 [0–94.5]	NA
Low risk of bias (3 data points)	3.3 [0–20.8]	71	78.4 [30.4–93.3]	0.68
*Streptococcus pyogenes* in RSV‐associated AOM				
Overall (3 data points)	0.3 [0–3.4]	106	0 [0–89.6]	0.632
Antibiotic_before_sampling (2 data points)	0.5 [0–4.5]	80	0	NA
Low risk of bias (3 data points)	0.3 [0–3.4]	106	0 [0–89.6]	0.632
*Chlamydophila pneumoniae* ^b^ in RSV‐associated AOM				
Overall (2 data points)	0 [0–4.7]	36	0	NA
Low risk of bias (1 data point)	0 [0–6.5]	26	NA	NA
*Mycoplasma pneumoniae* in RSV‐associated AOM^b^				
Overall (2 data points)	0 [0–4.6]	40	0	NA
Low risk of bias (1 data point)	0 [0–6.5]	26	NA	NA
*Legionella pneumophila* in RSV‐associated AOM^c^				
Overall (1 data point)	0 [0–6.5]	26	NA	NA
Low risk of bias (1 data point)	0 [0–6.5]	26	NA	NA
*Enterococcus faecalis* in RSV‐associated AOM^c^				
Overall (1 data point)	0 [0–12.8]	13	NA	NA
Antibiotic_before_sampling (1 data point)	0 [0–12.8]	13	NA	NA
Low risk of bias (1 data point)	0 [0–12.8]	13	NA	NA
*Escherichia coli* in RSV‐associated AOM^c^				
Overall (1 data point)	0 [0–12.8]	13	NA	NA
Antibiotic_before_sampling (1 data point)	0 [0–12.8]	13	NA	NA
Low risk of bias (1 data point)	0 [0–12.8]	13	NA	NA

Abbreviations: 95% CI, 95% confidence interval; Bacteria 1+, at least one bacterium; N, number.

^a^ *I* ^2^ describes the proportion of total variation in study estimates that is due to heterogeneity.

^b^ Only two data points.

^c^ Only one data point.

The subgroup analysis of bacterial co‐detections in children with RSV‐associated AOM showed higher proportions in inpatient settings for *S. pneumoniae* (48.1% [95% CI, 28.1–68.4]), *H. influenzae* (56.4% [95% CI, 40.5–71.7]), and *M. catarrhalis* (40.4% [95% CI, 30.3–50.9]) (Appendix S5). Seasonal studies reported higher proportion for at least one bacterium [Bacteria 1+] (87.5% [95% CI, 77.4–95.1]). Older children had higher proportions of *S. pneumoniae* (38.4% [95% CI, 22.3–55.8]), *H. influenzae* (51.4% [95% CI, 27.7–74.8]), and *M. catarrhalis* (22.1% [95% CI, 5.8–44.3]). Nasopharyngeal samples were associated with higher proportion for Bacteria 1+ (92.5% [95% CI, 83.5–98.3]), *S. pneumoniae* (58.7% [95% CI, 48.4–68.7]), *H. influenzae* (56.4% [95% CI, 40.5–71.7]), and *M. catarrhalis* (49.4% [95% CI, 24.5–74.5]). The use of PCR detection was associated with higher proportion for Bacteria 1+ (92.5% [95% CI, 83.5–98.3]), *S. pneumoniae* (58.5% [95% CI, 44.9–71.5]), and *M. catarrhalis* (62.3% [95% CI, 48.7–74.9]). Regional and economic disparities were evident, with subgroup categories represented by a low number of studies, making it difficult to draw meaningful conclusions.

## 4 Discussion

This is the first systematic review to provide a comprehensive assessment of the burden of RSV in children with AOM. We found that approximately one in six children with AOM had RSV infection identified. This proportion is substantial, considering the high global incidence of AOM. It underscores that RSV, beyond causing LRTI [3], also plays a significant role in AOM. Our analysis also showed that RSV‐associated AOM is often a polymicrobial infection, with about two‐thirds of cases involving a bacterial co‐detection. The most frequent co‐detected bacteria were non‐typeable *H. influenzae* , *S. pneumoniae* , and *M. catarrhalis* , which are typically the most common bacterial pathogens in AOM [40]. This finding aligns with the understanding that viral infections like RSV can predispose children to bacterial middle ear infection by causing inflammation and fluid build‐up in the middle ear [41]. In some instances, however, RSV alone may directly lead to AOM without any bacteria, as has been observed in certain clinical studies [42]. RSV infection can induce mucosal swelling and Eustachian tube dysfunction, which can create effusions in the middle ear and result in an infection even if no bacteria are initially present. Our findings support the concept that RSV is not only a coincidental bystander but can be a primary cause of AOM.

A finding of our subgroup analysis is the difference in RSV‐AOM burden between inpatient and outpatient settings. We noted a higher proportion of RSV in children with AOM among studies based in inpatient compared to outpatient settings. This is likely to reflect that children with more severe AOM or with complications (such as those requiring hospitalization or specialist referral) have a greater likelihood of RSV involvement [43]. Inpatients may represent more severe or complicated AOM cases, often following untreated episodes or concurrent respiratory bacterial co‐detections. In these cases, clinicians might perform more extensive diagnostic testing, such as tympanocentesis to obtain middle ear fluid and PCR tests. By contrast, the majority of AOM episodes occur in the outpatient setting, and many mild cases may never even be tested for viruses. Most AOM in the community is treated empirically and goes unreported in surveillance data. Our findings suggest that relying only on hospital data could overestimate the RSV association, whereas community‐based studies could underestimate it if they do not capture the full spectrum of disease severity, including mild, moderate, and severe cases. Therefore, both settings need to be studied to get a true picture of RSV's role in AOM.

The type of specimen collected for virus detection also influences the observed RSV proportions, which has implications for both research and clinical diagnosis. Nasopharyngeal samples yielded the highest bacterial proportion in RSV‐associated AOM (92.5% for bacteria 1+). These findings support the role of the nasopharynx as a key reservoir for bacterial colonization and viral infection in the pathogenesis of AOM. Bacterial species detected in the MEF may be preceded by nasopharyngeal colonization. We found that studies using combined nasopharyngeal and middle ear fluid sampling had higher detection of RSV (70.8%). This indicates that some cases might be missed if only one site is tested. In practice, nasopharyngeal swabs are easier and less invasive, and they catch most RSV infections because the virus typically replicates in the nasal passages. Occasionally, the virus might be present in the middle ear fluid even if the nasopharyngeal swab is negative, especially if the upper respiratory infection has resolved or if sampling was not optimal. Conversely, not all RSV‐positive nasopharyngeal cases will have virus detectable in the middle ear fluid at the time of exam. Using both samples increases sensitivity for virus detection (70.8%). For research purposes, our results highlight that differences in sampling protocols can lead to different proportion estimates, complicating direct comparisons between studies. Future studies aiming to assess viral causes of AOM should consider collecting both nasal and ear specimens when feasible. Comprehensive sampling strategies are essential to capture the full spectrum of viral and bacterial contributors to AOM.

Our review has several strengths. We searched English and Chinese language databases and included a broad range of studies from different countries and settings and applied rigorous methods to synthesize the data. We specifically looked at bacterial co‐detections, which provide a more nuanced understanding of RSV‐associated AOM than just virus proportion alone. Most of the studies we included were of high quality with low risk of bias, and we conducted multiple subgroup and sensitivity analyses to test the robustness of our findings. To our knowledge, this is the first meta‐analysis to quantify the proportion of RSV in AOM and to aggregate data on bacterial co‐detection in these cases, which is timely given the introduction of RSV vaccines and long‐acting monoclonal antibodies.

We also acknowledge important limitations. First, the heterogeneity across studies was high. The incidence of RSV in AOM varied widely, likely due to differences in study populations (e.g., community versus hospitalized), ages, seasons of data collection, and diagnostic methods. Although we attempted to account for some of these factors via subgroup analyses, the high degree of heterogeneity means the pooled estimate should be interpreted with caution as an average that may not apply equally in all contexts. Moreover, *I* ^2^ only captures statistical heterogeneity; there are other contributors to heterogeneity (case definitions, clinical specimens, diagnostic tests, and healthcare systems) that are not accounted for in this value. Second, many studies did not test for all pathogens. Over 60% of studies did not report any bacterial data. Third, there is a geographic bias in the available data: Nearly all studies were from high‐income countries, with a particular concentration in certain regions (Europe, North America, and a few in East Asia). We found virtually no data from low‐ and middle‐income countries or from regions in Africa or South Asia where the overall burden of AOM is known to be very high [1]. This gap limits the global generalizability of our findings. Children in low‐resource settings often experience more frequent AOM, and factors like malnutrition or limited access to care might influence the role of RSV and other pathogens. Finally, none of the studies examined the broader impacts of RSV‐associated AOM, such as days of pain, hearing loss, school/daycare absenteeism, or economic costs due to missed work by parents/carers. These outcomes are important for fully understanding the burden but were outside the scope of most research to date.

Recent real‐world data from the United States [44], the United Kingdom [45], and Europe and Latin America [46] show that maternal RSV immunization and long‐acting monoclonal antibodies are reducing RSV‐associated burden on hospital inpatient services. In March 2025, the WHO prequalified the maternal RSV vaccine ABRYSVO, representing an important milestone for introduction in low‐ and middle‐income countries [47]. Future research could explore the potential impact of these (as well as others in clinical development) RSV interventions on AOM incidence in children. Even if a fraction of AOM in children could be prevented by protecting them against RSV infection, this in turn could reduce unnecessary antibiotic use and health care visits. This adds to the overall value proposition for RSV immunization programs: Not only can they save lives and prevent hospitalizations from bronchiolitis and pneumonia, but they might also alleviate the burden of AOM in early childhood. Our findings reinforce the importance of integrated virus–bacteria prevention strategies [48]. For example, pneumococcal conjugate vaccines and Hib vaccines target the main bacterial causes of AOM; their widespread use has reduced AOM incidence [49]. If RSV vaccination is added, it might further reduce AOM directly and also indirectly by preventing the initial viral trigger for bacterial superinfection.

In conclusion, our systematic review and meta‐analysis show that RSV is a significant contributor to AOM in children < 5 years, and RSV‐associated AOM frequently involves concurrent bacterial pathogens. The burden of RSV in AOM is particularly evident in children, including severe cases that require hospital care. Strengthening surveillance in both outpatient and inpatient settings, especially in under‐studied low‐resource settings, will be important to capture the true global burden and strengthen the overall value proposition for RSV immunization programs.

## Author Contributions

**Sebastien Kenmoe:** conceptualization, methodology, writing – review and editing, formal analysis, data curation, writing – original draft, validation. **Harish Nair:** conceptualization, funding acquisition, writing – review and editing, supervision, project administration, formal analysis. **Marshall Dozier:** data curation, methodology, writing – review and editing. **Jingyi Liang:** data curation, methodology, formal analysis, writing – review and editing. **Ruth Jenkins:** data curation, methodology, writing – review and editing.

## Funding

This work was supported by Merck (MISP # 101907).

## Conflicts of Interest

H.N. reports grants from the Merck Sharp & Dohme, related to the submitted work; grants from WHO, the Innovative Medicines Initiative, the National Institute for Health Research, Pfizer, and Icosavax/AstraZeneca; and personal fees from the Gates Foundation, Pfizer, GSK, Merck, Icosavax/AstraZeneca, and Sanofi, outside the submitted work.

## Supporting information


**Appendix S1:** Supporting Information.
**Table S1:** Search strategy.
**Table S2:** Quality assessment.
**Table S3:** Characteristics of included studies.
**Figure S1:** Global map highlighting countries with data on bacterial codetection proportions in RSV‐infected children.
**Table S4:** Quality assessment results.
**Table S5:** Subgroup analyses of RSV proportion and bacterial codetections (overall and etiology‐specific) in children < 5 years with AOM.

## Data Availability

The data that support the findings of this study are available in the Supporting Information of this article.
