# Supplementary material for: The Burden of Respiratory Syncytial Virus in Children With Acute Otitis Media: A Systematic Review and Meta‐Analysis
Source: Influenza Other Respir Viruses. 2026 Feb 4;20(2):e70223. doi: 10.1111/irv.70223 (PMC12873495; doi:10.1111/irv.70223)
Supplement: Supplementary file 1 — Appendix S1: Supporting Information. Table S1: Search strategy. Table S2: Quality assessment. Table S3: Characteristics of included studies. Figure S1: Global map highlighting countries with data on bacterial codetection proportions in RSV‐infected children. Table S4: Quality assessment results. Table S5: Subgroup analyses of RSV proportion and bacterial codetections (overall and etiology‐specific) in children < 5 years with AOM. [file IRV-20-e70223-s001.docx]

Contents

[Appendix 1 0](#_Toc204166113)

[Supplementary table 1: Search strategy 0](#_Toc204166114)

[Supplementary table 2: Quality assessment 4](#_Toc204166115)

[Supplementary table 3: Characteristics of included studies 5](#_Toc204166116)

[Supplementary figure 1: Global map highlighting countries with data on bacterial codetection proportions in RSV-infected children 14](#_Toc204166117)

[Supplementary table 4: Quality assessment results 15](#_Toc204166118)

[Supplementary table 5: Subgroup analyses of RSV proportion and bacterial codetections (overall and etiology-specific) in children <5 years with AOM 17](#_Toc204166119)

[Reference 25](#_Toc204166120)

# Appendix 1

| **Section and Topic** | **Item #** | **Checklist item** | **Location where item is reported** |
| --- | --- | --- | --- |
| **TITLE** | | |  |
| Title | 1 | Identify the report as a systematic review. | 1 |
| **ABSTRACT** | | |  |
| Abstract | 2 | See the PRISMA 2020 for Abstracts checklist. | 1-2 |
| **INTRODUCTION** | | |  |
| Rationale | 3 | Describe the rationale for the review in the context of existing knowledge. | 2-3 |
| Objectives | 4 | Provide an explicit statement of the objective(s) or question(s) the review addresses. | 3 |
| **METHODS** | | |  |
| Eligibility criteria | 5 | Specify the inclusion and exclusion criteria for the review and how studies were grouped for the syntheses. | 4 |
| Information sources | 6 | Specify all databases, registers, websites, organisations, reference lists and other sources searched or consulted to identify studies. Specify the date when each source was last searched or consulted. | 3 |
| Search strategy | 7 | Present the full search strategies for all databases, registers and websites, including any filters and limits used. | 3 |
| Selection process | 8 | Specify the methods used to decide whether a study met the inclusion criteria of the review, including how many reviewers screened each record and each report retrieved, whether they worked independently, and if applicable, details of automation tools used in the process. | 4 |
| Data collection process | 9 | Specify the methods used to collect data from reports, including how many reviewers collected data from each report, whether they worked independently, any processes for obtaining or confirming data from study investigators, and if applicable, details of automation tools used in the process. | 4 |
| Data items | 10a | List and define all outcomes for which data were sought. Specify whether all results that were compatible with each outcome domain in each study were sought (e.g. for all measures, time points, analyses), and if not, the methods used to decide which results to collect. | 4 |
|  | 10b | List and define all other variables for which data were sought (e.g. participant and intervention characteristics, funding sources). Describe any assumptions made about any missing or unclear information. | 4 |
| Study risk of bias assessment | 11 | Specify the methods used to assess risk of bias in the included studies, including details of the tool(s) used, how many reviewers assessed each study and whether they worked independently, and if applicable, details of automation tools used in the process. | 5 |
| Effect measures | 12 | Specify for each outcome the effect measure(s) (e.g. risk ratio, mean difference) used in the synthesis or presentation of results. | 5-6 |
| Synthesis methods | 13a | Describe the processes used to decide which studies were eligible for each synthesis (e.g. tabulating the study intervention characteristics and comparing against the planned groups for each synthesis (item #5)). | 5-6 |
|  | 13b | Describe any methods required to prepare the data for presentation or synthesis, such as handling of missing summary statistics, or data conversions. | 5-6 |
|  | 13c | Describe any methods used to tabulate or visually display results of individual studies and syntheses. | 5-6 |
|  | 13d | Describe any methods used to synthesize results and provide a rationale for the choice(s). If meta-analysis was performed, describe the model(s), method(s) to identify the presence and extent of statistical heterogeneity, and software package(s) used. | 5-6 |
|  | 13e | Describe any methods used to explore possible causes of heterogeneity among study results (e.g. subgroup analysis, meta-regression). | 5-6 |
|  | 13f | Describe any sensitivity analyses conducted to assess robustness of the synthesized results. | 5-6 |
| Reporting bias assessment | 14 | Describe any methods used to assess risk of bias due to missing results in a synthesis (arising from reporting biases). | 6 |
| Certainty assessment | 15 | Describe any methods used to assess certainty (or confidence) in the body of evidence for an outcome. | 5 |
| **RESULTS** | | |  |
| Study selection | 16a | Describe the results of the search and selection process, from the number of records identified in the search to the number of studies included in the review, ideally using a flow diagram. | 6, Figure 1 |
|  | 16b | Cite studies that might appear to meet the inclusion criteria, but which were excluded, and explain why they were excluded. | Figure 1 |
| Study characteristics | 17 | Cite each included study and present its characteristics. | 6-7; Supplementary table 3 |
| Risk of bias in studies | 18 | Present assessments of risk of bias for each included study. | 7; Supplementary table 4 |
| Results of individual studies | 19 | For all outcomes, present, for each study: (a) summary statistics for each group (where appropriate) and (b) an effect estimate and its precision (e.g. confidence/credible interval), ideally using structured tables or plots. | Figure 2 |
| Results of syntheses | 20a | For each synthesis, briefly summarise the characteristics and risk of bias among contributing studies. | 7; Table 1. |
|  | 20b | Present results of all statistical syntheses conducted. If meta-analysis was done, present for each the summary estimate and its precision (e.g. confidence/credible interval) and measures of statistical heterogeneity. If comparing groups, describe the direction of the effect. | 7, Figure 2 |
|  | 20c | Present results of all investigations of possible causes of heterogeneity among study results. | 7; Table 1. |
|  | 20d | Present results of all sensitivity analyses conducted to assess the robustness of the synthesized results. | 7; Table 1. |
| Reporting biases | 21 | Present assessments of risk of bias due to missing results (arising from reporting biases) for each synthesis assessed. | Table 1. |
| Certainty of evidence | 22 | Present assessments of certainty (or confidence) in the body of evidence for each outcome assessed. | Table 1 |
| **DISCUSSION** | | |  |
| Discussion | 23a | Provide a general interpretation of the results in the context of other evidence. | 8-10 |
|  | 23b | Discuss any limitations of the evidence included in the review. | 11-12 |
|  | 23c | Discuss any limitations of the review processes used. | 12 |
|  | 23d | Discuss implications of the results for practice, policy, and future research. | 12-14 |
| **OTHER INFORMATION** | | |  |
| Registration and protocol | 24a | Provide registration information for the review, including register name and registration number, or state that the review was not registered. | PROSPERO database (CRD42024545528) |
|  | 24b | Indicate where the review protocol can be accessed, or state that a protocol was not prepared. | PROSPERO |
|  | 24c | Describe and explain any amendments to information provided at registration or in the protocol. | None |
| Support | 25 | Describe sources of financial or non-financial support for the review, and the role of the funders or sponsors in the review. | 14 |
| Competing interests | 26 | Declare any competing interests of review authors. | 14 |
| Availability of data, code and other materials | 27 | Report which of the following are publicly available and where they can be found: template data collection forms; data extracted from included studies; data used for all analyses; analytic code; any other materials used in the review. | Appendix |

*From:*  Page MJ, McKenzie JE, Bossuyt PM, Boutron I, Hoffmann TC, Mulrow CD, et al. The PRISMA 2020 statement: an updated guideline for reporting systematic reviews. BMJ 2021;372:n71. doi: 10.1136/bmj.n71. This work is licensed under CC BY 4.0. To view a copy of this license, visit <https://creativecommons.org/licenses/by/4.0/>

# Supplementary table 1: Search strategy

Embase Classic+Embase <1947 to 2024 June 10>

| 1 | acute respiratory tract disease/ or human respiratory syncytial virus/ or human respiratory syncytial virus a/ or exp infectious pneumonia/ or exp lower respiratory tract infection/ or exp pneumonia/ or respiratory syncytial virus infection/ or respiratory tract infection/ or respiratory tract disease/ | 760465 |
| --- | --- | --- |
| 2 | (acute respiratory infection* or bronchiolit* or lower respiratory infection* or lower respiratory tract infection* or pneumonia or respiratory infection* or respiratory disease* or respiratory syncytial virus* or ALRI or ALRTI or LRTI or RSV).mp. | 596019 |
| 3 | 1 or 2 | 882946 |
| 4 | exp child/ or childhood disease/ or exp infant disease/ or exp pediatrics/ | 5040382 |
| 5 | (babies or baby or boy* or child* or girlhood or girl? or infan* or kid? or neonat* or neo-nat* or newborn* or new-born* or paediatric* or peadiatric* or pediatric* or perinat* or preschool* or toddler*).mp. | 4990841 |
| 6 | (pediatric* or paediatric* or infan* or child* or young).in. | 2459437 |
| 7 | 4 or 5 or 6 | 7021353 |
| 8 | exp otitis media/ | 45048 |
| 9 | (otitis media or aom or ome or glue ear? or (middle and ear and (effusion or inflam* or infect*))).mp. [mp=title, abstract, heading word, drug trade name, original title, device manufacturer, drug manufacturer, device trade name, keyword heading word, floating subheading word, candidate term word] | 67872 |
| 10 | 8 or 9 | 67983 |
| 11 | 3 and 7 and 10 | 7282 |
| 12 | limit 11 to yr="1996 -Current" | 6110 |

Update search as above but with additional rows:

Embase Classic+Embase <1947 to 2024 September 19>

| 13 | limit 12 to dd=20240611-20240930 | 31 |
| --- | --- | --- |
| 14 | limit 12 to rd=20240611-20240930 | 63 |
| 15 | 13 or 14 | 94 |

Global Health <1973 to 2024 Week 22>

| 1 | bronchiolitis/ or bronchopneumonia/ or human respiratory syncytial virus/ or lower respiratory tract infections/ or pneumonia/ | 48269 |
| --- | --- | --- |
| 2 | (acute respiratory infection* or bronchiolit* or lower respiratory infection* or lower respiratory tract infection* or pneumonia or respiratory infection* or respiratory disease* or respiratory syncytial virus* or ALRI or ALRTI or LRTI or RSV).mp. [mp=abstract, title, original title, heading words, cabicodes words] | 207406 |
| 3 | 1 or 2 | 207455 |
| 4 | exp children/ or exp infants/ or childhood diseases/ or paediatrics/ | 493922 |
| 5 | (babies or baby or boy* or child* or girlhood or girl? or infan* or kid? or neonat* or neo-nat* or newborn* or new-born* or paediatric* or peadiatric* or pediatric* or perinat* or preschool* or toddler*).mp. | 643058 |
| 6 | (pediatric* or paediatric* or infan* or child* or young).in. | 218542 |
| 7 | 4 or 5 or 6 | 712679 |
| 8 | otitis media/ | 4396 |
| 9 | (otitis media or aom or ome or glue ear? or (middle and ear and (effusion or inflam* or infect*))).mp. | 8415 |
| 10 | 8 or 9 | 8415 |
| 11 | 3 and 7 and 10 | 1490 |
| 12 | limit 11 to yr="1996 -Current" | 1423 |

Global Health <1973 to 2024 Week 38>

Update search as above but with additional rows:

| 13 | limit 12 to rd=20240611-20240930 | 11 |
| --- | --- | --- |
| 14 | limit 12 to up=20240611-20240930 | 12 |
| 15 | 13 or 14 | 12 |

Ovid MEDLINE(R) ALL <1946 to June 10, 2024>

| 1 | bronchiolitis/ or bronchiolitis, viral/ or Bronchopneumonia/ or pneumonia/ or pneumonia, viral/ or Respiratory Syncytial Virus Infections/ or respiratory syncytial viruses/ or Respiratory Syncytial Virus, Human/ or respiratory tract infections/ or Respiratory Tract Diseases/ | 182472 |
| --- | --- | --- |
| 2 | (acute respiratory infection* or bronchiolit* or lower respiratory infection* or lower respiratory tract infection* or pneumonia or respiratory infection* or respiratory disease* or respiratory syncytial virus* or ALRI or ALRTI or LRTI or RSV).mp. | 330796 |
| 3 | 1 or 2 | 373274 |
| 4 | exp child/ or exp infant/ or Infant Health/ or pediatrics/ | 2870200 |
| 5 | (babies or baby or boy* or child* or girlhood or girl? or infan* or kid? or neonat* or neo-nat* or newborn* or new-born* or paediatric* or peadiatric* or pediatric* or perinat* or preschool* or toddler*).mp. | 3894933 |
| 6 | (pediatric* or paediatric* or infan* or child* or young).in. | 1670809 |
| 7 | 4 or 5 or 6 | 4650744 |
| 8 | exp Otitis Media/ | 26115 |
| 9 | (otitis media or aom or ome or glue ear? or (middle and ear and (effusion or inflam* or infect*))).mp. | 46819 |
| 10 | 8 or 9 | 48013 |
| 11 | 3 and 7 and 10 | 3276 |
| 12 | limit 11 to yr="1996 -Current" | 2343 |

Update search as above but with additional rows:

Ovid MEDLINE(R) ALL <1946 to September 19, 2024>

| 13 | limit 12 to dt=20240611-20240930 | 26 |
| --- | --- | --- |
| 14 | limit 12 to rd=20240611-20240930 | 97 |
| 15 | 13 or 14 | 97 |

Web of Science

- WOS.IC: 1993 to 2024

- WOS.CCR: 1985 to 2024

- WOS.SCI: 1900 to 2024

- WOS.AHCI: 1975 to 2024

- WOS.BHCI: 2005 to 2024

- WOS.BSCI: 2005 to 2024

- WOS.ESCI: 2015 to 2024

- WOS.ISTP: 1990 to 2024

- WOS.SSCI: 1900 to 2024

- WOS.ISSHP: 1990 to 2024

| 1 | TS=("acute respiratory infection*" or bronchiolit* or "lower respiratory infection*" or "lower respiratory tract infection*" or pneumonia or "respiratory infection*" or "respiratory disease*" or "respiratory syncytial virus*" or ALRI or ALRTI or LRTI or RSV) | 292088 |
| --- | --- | --- |
| 2 | TS=(babies or baby or boy* or child* or girlhood or girl? or infan* or kid? or neonat* or neo-nat* or newborn* or new-born* or paediatric* or peadiatric* or pediatric* or perinat* or preschool* or toddler*) | 3634051 |
| 3 | OO=(pediatric* or paediatric* or infan* or child* or young) | 1408653 |
| 4 | #2 OR #3 | 4374728 |
| 5 | TS=(otitis media or aom or ome or glue ear? or (middle and ear and (effusion or inflam* or infect*))) | 49693 |
| 6 | #1 AND #4 AND #5 | 2441 |
| 7 | #6 Timespan: 1996-01-01 to 2024-06-10 | 2220 |

[update search: as above, with index date limit from 2024-06-10 to 2024-09-20]

Scopus

TITLE-ABS-KEY ( ( "acute respiratory infection*" OR bronchiolit* OR "lower respiratory infection*" OR "lower respiratory tract infection*" OR pneumonia OR "respiratory infection*" OR "respiratory disease*" OR "respiratory syncytial virus*" OR alri OR alrti OR lrti OR rsv ) )

AND

( TITLE-ABS-KEY ( babies OR baby OR boy* OR child* OR girlhood OR girl* OR infan* OR kid* OR neonat* OR neo-nat* OR newborn* OR new-born* OR paediatric* OR peadiatric* OR pediatric* OR perinat* OR preschool* OR toddler* ) OR AFFILORG ( pediatric* OR paediatric* OR infan* OR child* OR young ) )

AND

( TITLE-ABS-KEY ( otitis AND media OR aom OR ome OR glue AND ear* OR ( middle AND ear AND ( effusion OR inflam* OR infect* ) ) ) )

AND

PUBYEAR > 1995 AND PUBYEAR < 2025

[update search on September 20, 2024: as above, with Year limit 2024-2024]

**CNKI**

[Subject:] (毛细支气管炎 or 肺炎 or 呼吸道合胞病毒感染 or 呼吸道合胞病毒 or 呼吸道感染 or 呼吸道疾病 or 急性呼吸道感染or 下呼吸道感染or ALRI or ALRTI or LRTI or RSV)

AND

[Subject:] (儿童 or 婴儿 or 婴儿健康 or 儿科 or 新生儿 or 学龄前儿童 or 学龄前幼儿)

AND

[Subject:] (中耳炎 or 渗出性中耳炎 or OME or AOM)

Publication time: 1996 to 2024-September

**321** records retrieved on 7^th^ Feburary 2025.

Search term:
*(SU%=毛细支气管炎 or SU%=肺炎 or SU%=呼吸道合胞病毒感染 or SU%=呼吸道合胞病毒 or SU%=呼吸道感染 or SU%=呼吸道疾病 or SU%= 急性呼吸道感染 or SU%=下呼吸道感染 or SU%=ALRI or SU%=ALRTI or SU%=LRTI or SU%=RSV)*

*AND (SU%=儿童 or SU%=婴儿 or SU%=婴儿健康 or SU%=儿科 or SU%=新生儿 or SU%=学龄前儿童 or SU%=学龄前幼儿)*

*AND (SU%=中耳炎 or SU%=渗出性中耳炎 or SU%=OME or SU%=AOM)*

**Wanfang**

[Subject:] (毛细支气管炎 or 肺炎 or 呼吸道合胞病毒感染 or 呼吸道合胞病毒 or 呼吸道感染 or 呼吸道疾病 or 急性呼吸道感染or 下呼吸道感染or ALRI or ALRTI or LRTI or RSV)

AND

[Subject:] (儿童 or 婴儿 or 婴儿健康 or 儿科 or 新生儿 or 学龄前儿童 or 学龄前幼儿)

AND

[Subject:] (中耳炎 or 渗出性中耳炎 or OME or AOM)

Publication time: 1996 to 2024

**704** records retrieved on 7^th^ Feburary 2025.

Search term:
*(主题:(毛细支气管炎 or 肺炎 or 呼吸道合胞病毒感染 or 呼吸道合胞病毒 or 呼吸道感染 or 呼吸道疾病 or 急性呼吸道感染 or下呼吸道感染or ALRI or ALRTI or LRTI or RSV))*

*and (主题:(儿童 or 婴儿 or 婴儿健康 or 儿科 or 新生儿 or 学龄前儿童 or 学龄前幼儿))*

*and (主题:(中耳炎 or 渗出性中耳炎 or OME or AOM))*

**Chongqing VIP**

[Subject:] (毛细支气管炎 or 肺炎 or 呼吸道合胞病毒感染 or 呼吸道合胞病毒 or 呼吸道感染 or 呼吸道疾病 or 急性呼吸道感染or 下呼吸道感染or ALRI or ALRTI or LRTI or RSV)

AND

[Subject:] (儿童 or 婴儿 or 婴儿健康 or 儿科 or 新生儿 or 学龄前儿童 or 学龄前幼儿)

AND

[Subject:] (中耳炎 or 渗出性中耳炎 or OME or AOM)

Publication time: 1996 to 2024

**72** records retrieved on 7^th^ Feburary 2025.

Search term:
*(M=(毛细支气管炎 or 肺炎 or 呼吸道合胞病毒感染 or 呼吸道合胞病毒 or 呼吸道感染 or 呼吸道疾病 or急性呼吸道感染 or下呼吸道感染or ALRI or ALRTI or LRTI or RSV))*

*and (M=(儿童 or 婴儿 or 婴儿健康 or 儿科 or 新生儿 or 学龄前儿童 or 学龄前幼儿))*

*and (M=(中耳炎 or 渗出性中耳炎 or OME or AOM))*

# Supplementary table 2: Quality assessment

| Variable Name | Description | Scores |
| --- | --- | --- |
| Study site representativeness | Whether the study sites encompass diverse areas representing the national population | Yes (1) /No (0) /Unclear (0) |
| Inclusion/exclusion criteria | Detailed list of inclusion and exclusion criteria | Yes (1) /No (0) /Unclear (0) |
| Setting description | Types of clinical or community settings where data were collected | Yes (1) /No (0) /Unclear (0) |
| Participant characteristics | Comprehensive demographic and clinical characteristics of the subjects | Yes (1) /No (0) /Unclear (0) |
| AOM definitions | Definitions used for otitis media | Yes (1) /No (0) /Unclear (0) |
| RSV assay details | Specific details about the RSV assay (e.g., PCR, viral culture, DFA) | Yes (1) /No (0) /Unclear (0) |
| Study period dates | Start and end dates of the study period, evidence that the study was uninterrupted, or justification for any breaks in the study period | Yes (1) /No (0) /Unclear (0) |
| Antibiotic use details | Specific details on the type or duration of antibiotic use prior to sample collection | Yes (1) /No (0) /Unclear (0) |
| Statistical methods | Detailed description of statistical tests and models used, Clarity on whether all relevant factors were included in the analyses, such as adjustments for potential confounders | Yes (1) /No (0) /Unclear (0) |

# Supplementary table 3: Characteristics of included studies

| **Authors** | **Study Design** | **Setting details** | **Location** | **Study period** | **Age range** | **Antibiotic status prior to sample collection (percentage)** | **Antibiotic administration status (percentage)** | **Vaccination status** | **Population inclusion criteria** | **Otitis media types** | **Otitis media diagnostic** | **AOM duration** | **Clinical specimen for RSV test** | **RSV diagnostic test** | **Clinical specimen for Bacterial test** | **Bacterial diagnostic test** | **Data analyses** |
| --- | --- | --- | --- | --- | --- | --- | --- | --- | --- | --- | --- | --- | --- | --- | --- | --- | --- |
| (Andrade et al., 1998) | Cohort (Prospetive) | Outpatient | United States of America (Pittsburgh) | Dec/1995-Jan/1997 | 2-24 months | 0 | Not specified | Not specified | Children aged 2-24 months seen in primary care or ED (Pittsburgh) with diagnosed bronchiolitis. | AOM, OME | pneumatic otoscopy | Not specified | nasal washings; middle-ear aspirates | ELISA; PCR | Not done | Not done | Counts, percentages, means |
| (Aymard et al., 1997) | Cross-sectional (Retroprospective) | Outpatient | France (Bordeaux and Lyon) | Oct/1995-Mar/1996 | 0-60 months | Not specified | 47 | Not specified | Children in Bordeaux and Lyon with otitis or rhinopharyngitis, providing nasal/pharyngeal samples. | OM; AOM | Not specified | Not specified | fosses nasales/cavum | immunofluorescence; PCR | fosses nasales | PCR | Counts, percentages |
| (Bulut et al., 2007) | Cohort (Prospetive) | Outpatient | Türkiye | Mar/2003-Dec/2004 | 6-24 months | 0 | Amoxicillin-clavulunate (100) | Not specified | Children 6-24 months with AOM signs; no recent antibiotics; no chronic OM; parental consent. | AOM | pediatrician, otorhinolaryngology specialist | Not specified | MEF | Culture; PCR | MEF | Culture | Chi-square test, Spearman’s rank order correlation coefficient, and Fisher exact Chi-square test. |
| (Chonmaitree et al., 2008) | Cohort (Prospetive) | Household/ Outpatient | United States of America (Texas) | Jan/2003-Mar/2006 | 6-36 months | Not specified | Not specified | Not specified | Children followed for 1 year for symptomatic URIs, capturing cases complicated by OM. | AOM, OME | study physician, pneumatic otoscopy and/or tympanometry | Not specified | Nasal swab; NP secretions | viral culture; EIA; PCR | Not done | Not done | General estimating equations approach accounting for the multiple episodes of correlated data from each child. Binomial distribution, logit link function, and AR(1) correlation structure. Rate ratios, and Pearson x2 statistics. |
| (Diaz et al., 2018) | Cross-sectional (Retrospective) | Inpatient/ Outpatient | United States of America | 2010-2014 | 0-24 months | 40 | Not specified | Not specified | Previously healthy children <2 years with acute RSV infection (2010-2014). | AOM | Not specified | Not specified | NP swabs | PCR | NP swabs | PCR | Counts, percentages, medians, Chi-square test or Fisher’s exact test, Mann-Whitney U test |
| (Gomaa et al., 2012) | Cohort (Prospetive) | Inpatient | Saudi Arabia (Minia) | 2009 | 3-18 months | 0 | Not specified | Not specified | Children 3-18 months hospitalized with acute bronchiolitis. | AOM | tympanometry | 14 days | middle ear aspirate | culture; ELISA | middle ear aspirate | culture | Counts, percentages |
| (Kafetzis et al., 2003) | Cohort (Prospetive) | Inpatient | Greece (Athens) | Jan/1999-Jun/2000 | 0-24 months | Not specified | Not specified | Not specified | Children 2 weeks-2 years hospitalized for respiratory distress during RSV season, without RSV prophylaxis. | AOM | ear, nose and throat specialist | Not specified | NP washings | direct fluorescence assay | Not done | Not done | Chi-square test and Fisher's exact test |
| (Kleemola et al., 2006) | Cohort (Prospetive) | Outpatient | Finland (Tampere) | Apr/1994-Mar/1999 | 2-24 months | 0 | Not specified | Not specified | Children from the FinOM Cohort (329 healthy) and 198 controls from the FinOM vaccine trial. | AOM | study doctor | 30 days | MEF; NP aspirates | Culture; TRFIA | MEF | Culture | Counts, percentages |
| (Kotikoski et al., 2002) | Cohort (Prospetive) | Outpatient | Finland (Turku) | Oct/1997-Mar/1999 | 7-24 months | Not specified | Not specified | PCV (66.6) | Children 7-23 months followed to age 24 months in a cohort. | AOM | study physicians, pneumatic otoscopy | Not specified | MEF; NP aspirate | culture; TRFIA | MEF | culture | Chi-square test |
| (Kuczborska et al., 2021) | Cross-sectional (Retrospective) | Inpatient | Poland (Warsaw) | Jan/2018-Mar/2020 | 0-18 months | Not specified | More often in the group with non-RSV infection (P = 0.001) | Not specified | Children <18 months with clinical and imaging-confirmed ALRI. | AOM | Not specified | Not specified | nasal swab | immunochromatographic test | Not done | Not done | Mann-Whitney U-test, chi-square test |
| (Madhi et al., 2015) | Cohort (Prospetive) | Outpatient | South Africa (Soweto) | May/2009-Apr/2010 | 4-58 months | 0 | Not specified | ≥1 dose PCV (16.1), 3 doses PCV (27.5); 4 doses (2.5). No significant differences in the prevalence of S. pneumoniae between PCV-vaccinated (10.0%; 95% CI: 2.8–23.7) and unvaccinated children [22.3% (95% CI: 17.0–28.4); P = 0.08] or in the prevalence of H. influenzae [35.0% (95% CI: 20.6−51.7) vs. 30.0% (95% CI: 24.0−36.5), respectively; P = 0.53]. S. pneumoniae serotypes observed in vaccinated children were 14, 18C, 21 and 6A (each accounting for one episode). Serotypes 1, 6B, 14, 15B, 15C, 16, 19A, 19F, 21, 23F, 3, 4, 5, 6A, 6B, 9N and 9V (each accounting for at least 1 episode) were detected in unvaccinated children. No statistically significant difference was detected in the prevalence of PCV7-serotypes between PCV-vaccinated [5.0% (95% CI: 0.6–16.9)] and unvaccinated children [12.7% (95% CI: 8.6–17.9); P = 0.28). Of 80 H. influenzae-positive episodes, 65 in the PCV-unvaccinated group and 14 in the PCV-vaccinated group were NTHi. | Children 3 months-<5 years with AOM confirmed by ENT specialist. | AOM | ear–nose–throat specialist | 30 days | MEF; NP aspirates | Culture; immunofluorescence assay and PCR | MEF | Culture | Counts, percentages, 95% confidence interval |
| (Marom et al., 2017) | Cohort (Retrospective) | Inpatient | Israel | Jan/2010-Dec/2015 | 0-12 months | 28 | AOM+ (99); Change of Abx (17.8) | ≥2 PCV13 dose (>95) | Infants ≤1 year hospitalized with AOM (Israel, 2010-2015). | OM, AOM | ICD-9 | Not specified | nasal secretions | Unclear/Not reported | Not done | Not done | Percentages, medians and interquartile ranges, Mann-Whitney tests, Fisher's exact and Chi-square tests. |
| (Monobe et al., 2003) | Cross-sectional (Prospetive) | Outpatient | Japan | 1999-2001 | 0-24 months | 0 | Cefditoren pivoxil (100) | Not specified | Children 5-24 months with AOM (Japan, 1999-2001). | AOM | pneumatic otoscopic | Persistent MEE, Early recurrence, Recurrence AOM | MEF | PCR | Not done | Not done | Proportions, median, Mann/Whitney U-test. |
| (Nokso-Koivisto et al., 2004) | Cohort (Prospetive) | Outpatient | Finland (Tampere) | Apr/1994-Aug/1998 | 2-24 months | Not specified | Not specified | Not specified | Children from FinOM Cohort and FinOM Vaccine Trial (healthy or vaccinated). | AOM | study doctor, myringotomy | Not specified | MEF; NP aspirate | TRFIA | Not done | Not done | Counts, percentages, rates (per person-year) |
| (Papan et al., 2020) | Cohort (Retrospective) | Inpatient | Germany (Heidelberg) | Apr/2014-Apr/2018 | 0-24 months | Not specified | RSV+ (26.4) | Not specified | Children <2 years hospitalized with RSV or influenza (2014-2018). | AOM | Not specified | Not specified | NP swab | rapid point-of-care test; PCR | Not done | Not done | Mann-Whitney U test, Kruskal-Wallis test and one-way analysis of variance, and logistic regressions. |
| (Pettigrew et al., 2011) | Cohort (Prospetive) | Outpatient | United States of America (Texas) | Jan/2003-Mar/2007 | 6-36 months | 17.2 | Not specified | PCV7 (>99) | Prospective cohort of children with AOM complicating symptomatic URI. | AOM | pneumatic otoscopy and/or tympanometry | Not specified | NP secretion specimens | PCR | Not done | Not done | Logistic regression with generalized estimating equations (GEE) and an autoregressive correlation structure (AR1). All models were adjusted for age, ethnicity, daycare attendance, breastfed, exposure to environmental tobacco smoke, and antibiotic use. |
| (Pitkaranta et al., 1998) | Cross-sectional (Prospetive) | Inpatient | Finland (Helsinki ) | 1993-1994 | 0-24 months | Not specified | Not specified | Not specified | Children 0-24 months undergoing tympanostomy/adenoidectomy for persistent OME. | OME | Physicians (pneumatic otoscopy), tympanostomy | Not specified | MEE samples | PCR | Not done | Not done | Counts, percentages |
| (Raty et al., 2004) | Cohort (Prospetive) | Outpatient | Finland (Tampere) | Apr/1994-Jul/1997 | 0-24 months | Not specified | Not specified | Not specified | Children enrolled at 2 months and followed to 2 years. | AOM | study doctor | Not specified | NP aspirate; MEF; acute and convalescent phase sera | EIA (IgG); TRFIA | Not done | Not done | Counts, percentages |
| (Rosenblüt et al., 2001) | Cross-sectional (Prospetive) | Outpatient | Chile (Santiago) | Jul/1998-Jun/1999 | 3-48 months | 0 | Not specified | Not specified | Children ≥3 months with presumptive AOM referred from ED (Chile). | AOM | ED pediatricians (otomicroscopic examination and/or had tympanostomy) | Not specified | NP aspirate; MEF | indirect immunofluorescence; Culture | Not done | Not done | Medians and ranges, frequencies, percents, chi square test |
| (Ruohola et al., 2013) | Cohort (Prospetive) | Outpatient | Finland (Southwest) | 2006-2009 | 6-35 months | Not specified | Not specified | ≥1 dose of PCV (2); ≥1 dose of HiB (100) | Children 6-35 months with acute symptoms suggestive of AOM. | AOM | Pneumatic otoscopic | Not specified | NP samples | TRFIA; PCR | Not done | Not done | Means, T test, proportions, Chi-square test, logistic regression |
| (Sawada et al., 2019) | Cross-sectional (Prospetive) | Outpatient | Japan (Kochi) | Jan/2016-Dec/2017 | 4-36 months | Not specified | Not specified | PCV13 (100) | Children with AOM requiring paracentesis, prolonged fever/earache, or treatment failure (Japan). | AOM | Otolaryngologist (otoscopic) | Not specified | NP aspirates and MEF | culture; PCR | MEF samples | culture; PCR | Counts, percentages |
| (Toivonen et al., 2020) | Cohort (Prospetive) | Household/ Outpatient | Finland (Southwest) | Jan/2008-Apr/2010 | 0-24 months | Not specified | Not specified | Not specified | Children born in Southwest Finland (2008-2010) recruited in STEPS birth cohort. | AOM | Study physician (Pneumatic otoscopy and tympanometry) | Not specified | Nasal swab samples | PCR; antigen test | Not done | Not done | Chi-square or Fisher’s exact test, Wilcoxon–Mann–Whitney test, rates and 95% CIs per 100 children using generalized linear models. Overdispersed outcome counts using negative binomial distribution and log link. For low or non-overdispersed outcome counts, Poisson distribution was used. Number of RSV-associated outcomes, Hospitalization rates. |
| (Tomochika et al., 2009) | Case control (Retrospective) | Inpatient | Japan (Yamaguchi) | Jan/2004-Dec/2007 | 6-35 months | Not specified | 100 | Not specified | Children 6-35 months hospitalized with AOM (Japan, 2004-2007). | AOM | otoscopy by a pediatrician and then otolaryngologist | Not specified | NP swab specimens | culture; immunochromatography test | NP | culture | T-test and Chi-square test |
| (Uitti et al., 2015) | Cohort (Prospetive) | Household/ Outpatient | Finland (Turku) | 2006-2008 | 6-35 months | Not specified | Not specified | ≥1 dose of HiB (100); ≥1 dose of PCV (2) | Children 6-35 months presenting with acute infection symptoms and suspected AOM. | AOM | Otoscopy | Not specified | NP samples | TRFIA | Not done | Not done | χ2 test, multivariable logistic regression models adjusted by age, diagnosis of AOM and use of antipyretics. |
| (Vesa et al., 2001) | Cohort (Prospetive) | Outpatient | Finland (Tampere) | Apr/1994-Jul/1995 | 2-24 months | Not specified | Not specified | Not specified | Healthy infants in one Finnish clinic area born 1994-1995 offered participation. | AOM | study doctor (general practitioner), pneumatic otoscopy, myringotomy | Not specified | NP aspirates | solid phase immunoassay and TRFIA | Not done | Not done | Counts, percentages |
| (Wiertsema et al., 2011) | Cohort (Prospetive) | Inpatient | Australia (Perth) | Nov/2007-May/2009 | 6-36 months | Not specified | Not specified | Not specified | Children 6-36 months with recurrent AOM (GROMIT study). | rAOM | Not specified | Not specified | NP swabs; MEE | culture; PCR | NP swabs; MEE | culture | Mann–Whitney analyses, Pearson chi-square analyses, Binary logistic regression |
| (Yatsyshina et al., 2016) | Cross-sectional (Retrospective) | Inpatient | Russian Federation (Moscow) | Aug/2011-Apr/2013 | 0-60 months | 0 | Not specified | Not specified | Children <5 years with AOM diagnosed by ENT and MEF specimen collected (Moscow, 2011-2013). | AOM | otolaryngologists | Not specified | MEF | PCR | Not done | Not done | Mann–Whitney test, sensitivity, specificity, and positive and negative predictive values, Cramer's V test |

AOM: Acute Otitis Media; CI: Confidence Interval; EIA: Enzyme Immunoassay; ED: Emergency Department; ELISA: Enzyme-Linked Immunosorbent Assay; HiB: Haemophilus influenzae type B; ICD-9: International Classification of Diseases, 9th Revision; IgG: Immunoglobulin G; MEE: Middle Ear Effusion; MEF: Middle Ear Fluid; NP: Nasopharyngeal; OME: Otitis Media with Effusion; OM: Otitis Media; PCR: Polymerase Chain Reaction; PCV: Pneumococcal Conjugate Vaccine; PCV7: 7-valent Pneumococcal Conjugate Vaccine; PCV13: 13-valent Pneumococcal Conjugate Vaccine; rAOM: Recurrent Acute Otitis Media; RSV: Respiratory Syncytial Virus; TRFIA: Time-Resolved Fluoroimmunoassay.

# Supplementary figure 1: Global map highlighting countries with data on bacterial codetection proportions in RSV-infected children


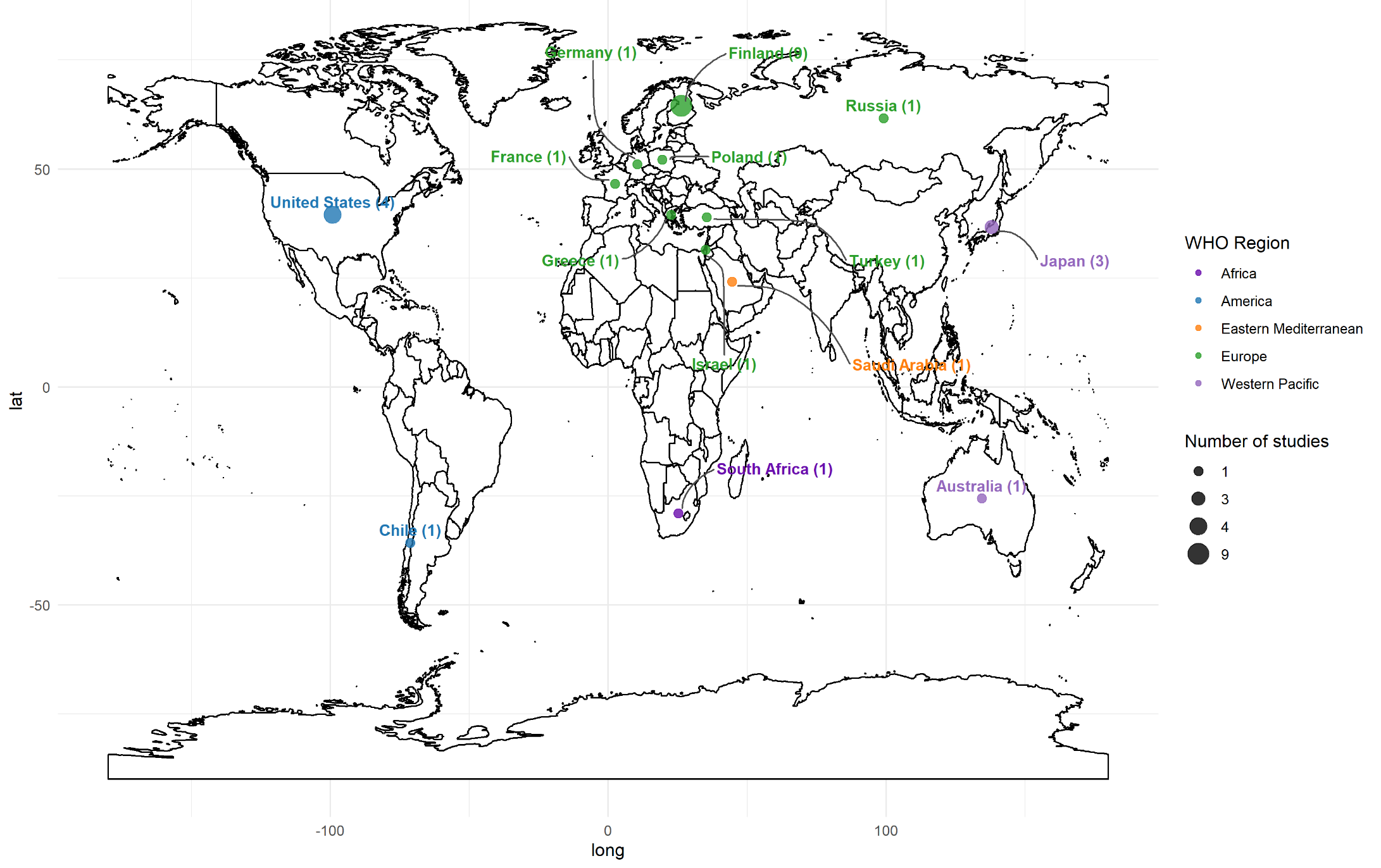


# Supplementary table 4: Quality assessment results

| Author | Year (Year of publication) | Study site representativeness | Sampling method | Inclusion exclusion criteria | Setting description | Participant characteristics | OM definitions | Detection assay details | Study period dates | Antibiotic use details | Statistical methods | Risk of bias |
| --- | --- | --- | --- | --- | --- | --- | --- | --- | --- | --- | --- | --- |
| (Andrade et al., 1998) | 1998 | No | No | Yes | Yes | Yes | Yes | Yes | Yes | Yes | Yes | Low risk of bias |
| (Aymard et al., 1997) | 1997 | No | No | Yes | Yes | Yes | No | Yes | No | Yes | Yes | Moderate risk of bias |
| (Bulut et al., 2007) | 2007 | No | No | Yes | Yes | Yes | Yes | Yes | No | Yes | Yes | Low risk of bias |
| (Chonmaitree et al., 2008) | 2008 | No | No | Yes | Yes | Yes | Yes | Yes | Yes | No | Yes | Low risk of bias |
| (Diaz et al., 2018) | 2018 | Unclear | No | Yes | Unclear | Yes | Yes | Unclear | Yes | Yes | Yes | Moderate risk of bias |
| (Gomaa et al., 2012) | 2012 | No | No | Yes | Yes | Yes | Yes | Yes | No | Yes | Yes | Low risk of bias |
| (Kafetzis et al., 2003) | 2003 | No | No | Yes | Yes | Yes | Yes | Yes | No | No | Yes | Moderate risk of bias |
| (Kleemola et al., 2006) | 2006 | Yes | Yes | Yes | Yes | Yes | Yes | Yes | Yes | Yes | Yes | Low risk of bias |
| (Kotikoski et al., 2002) | 2002 | No | Yes | Yes | Yes | Yes | Yes | Yes | Yes | No | Yes | Low risk of bias |
| (Kuczborska et al., 2021) | 2021 | No | No | Yes | Yes | Yes | No | Yes | Yes | Yes | Yes | Low risk of bias |
| (Madhi et al., 2015) | 2015 | No | No | Yes | Yes | Yes | Yes | Yes | Yes | Yes | Yes | Low risk of bias |
| (Marom et al., 2017) | 2017 | Yes | No | Yes | Yes | Yes | Yes | Unclear | Yes | Yes | Yes | Low risk of bias |
| (Monobe et al., 2003) | 2003 | No | Yes | Yes | Yes | Yes | Yes | Yes | No | Yes | Yes | Low risk of bias |
| (Nokso-Koivisto et al., 2004) | 2004 | No | Yes | Yes | Yes | Yes | Yes | Yes | Yes | No | Yes | Low risk of bias |
| (Papan et al., 2020) | 2020 | Yes | No | Yes | Yes | Yes | Yes | Yes | Yes | Yes | Yes | Low risk of bias |
| (Pettigrew et al., 2011) | 2011 | No | No | Yes | Yes | Yes | Yes | Yes | Yes | Yes | Yes | Low risk of bias |
| (Pitkaranta et al., 1998) | 1998 | No | No | Yes | Yes | Yes | Yes | Yes | Yes | No | Yes | Low risk of bias |
| (Raty et al., 2004) | 2004 | No | No | Yes | Yes | Yes | No | Yes | Yes | No | Yes | Moderate risk of bias |
| (Rosenblüt et al., 2001) | 2001 | Yes | No | Yes | Yes | Yes | Yes | Yes | Yes | Yes | Yes | Low risk of bias |
| (Ruohola et al., 2013) | 2013 | No | No | Yes | Yes | Yes | Yes | Yes | Yes | No | Yes | Low risk of bias |
| (Sawada et al., 2019) | 2019 | No | No | Yes | Yes | Yes | Yes | Yes | Yes | No | Yes | Low risk of bias |
| (Toivonen et al., 2020) | 2020 | No | No | Yes | Yes | Yes | Yes | Yes | Yes | Yes | Yes | Low risk of bias |
| (Tomochika et al., 2009) | 2009 | No | No | Yes | Yes | Yes | Yes | Yes | Yes | Yes | Yes | Low risk of bias |
| (Uitti et al., 2015) | 2015 | No | No | Yes | Yes | Yes | Yes | Yes | Yes | No | Yes | Low risk of bias |
| (Vesa et al., 2001) | 2001 | Yes | No | Yes | Yes | Yes | Yes | Yes | Yes | Unclear | Yes | Low risk of bias |
| (Wiertsema et al., 2011) | 2011 | No | No | Yes | Yes | Yes | Yes | Yes | Yes | Unclear | Yes | Low risk of bias |
| (Yatsyshina et al., 2016) | 2016 | No | No | Yes | Yes | Yes | Yes | Yes | Yes | Yes | Yes | Low risk of bias |

# Supplementary table 5: Subgroup analyses of RSV proportion and bacterial codetections (overall and etiology-specific) in children <5 years with AOM

|  | **RSV in AOM** |  |  |  | **Bacteria 1+ in RSV-associated AOM** |  |  |  | **Streptococcus pneumoniae in RSV-associated AOM** |  |  |  | **Haemophilus influenzae in RSV-associated AOM** |  |  |  | **Moraxella catarrhalis in RSV-associated AOM** |  |  |  |
| --- | --- | --- | --- | --- | --- | --- | --- | --- | --- | --- | --- | --- | --- | --- | --- | --- | --- | --- | --- | --- |
|  | **Prevalence (95% CI)** | **N Data points** | **N Cases** | **I² [95% CI]** | **Prevalence (95% CI)** | **N Data points** | **N Cases** | **I² [95% CI]** | **Prevalence (95% CI)** | **N Data points** | **N Cases** | **I² [95% CI]** | **Prevalence (95% CI)** | **N Data points** | **N Cases** | **I² [95% CI]** | **Prevalence (95% CI)** | **N Data points** | **N Cases** | **I² [95% CI]** |
| **Settings** |  |  |  |  |  |  |  |  |  |  |  |  |  |  |  |  |  |  |  |  |
| Inpatient | 26.4[10.2-46.7] | 7 | 754 | 1[1-1] | 87.5[77.4-95.1] | 1 | 56 | NA | 48.1[28.1-68.4] | 2 | 89 | 0.7[0-0.9] | 56.4[40.5-71.7] | 1 | 39 | NA | 40.4[30.3-50.9] | 2 | 89 | 0 |
| Outpatient | 14.9[8.8-22.2] | 15 | 6020 | 0.9[0.9-0.9] | 14.9[7.3-24.6] | 1 | 67 | NA | 15.4[3-33.4] | 5 | 170 | 0.9[0.7-0.9] | 19.1[1.9-45.7] | 5 | 170 | 0.9[0.8-1] | 6.6[2.4-12.4] | 5 | 170 | 0.2[0-0.8] |
| Household/Outpatient | 8.7[5-13.4] | 3 | 1568 | 0.9[0.7-1] | -- | -- | -- | -- | -- | -- | -- | -- | -- | -- | -- | -- | -- | -- | -- | -- |
| Inpatient/Oupatient | -- | -- | -- | -- | 92.5[83.5-98.3] | 1 | 53 | NA | 58.5[44.9-71.5] | 1 | 53 | NA | -- | -- | -- | -- | 62.3[48.7-74.9] | 1 | 53 | NA |
| **Country** |  |  |  |  |  |  |  |  |  |  |  |  |  |  |  |  |  |  |  |  |
| France | 9.9[5.5-15.5] | 1 | 141 | NA | -- | -- | -- | -- | -- | -- | -- | -- | -- | -- | -- | -- | -- | -- | -- | -- |
| Turkey | 26[14.7-39.2] | 1 | 50 | NA | -- | -- | -- | -- | 23.1[3.6-50.4] | 1 | 13 | NA | 7.7[0-30.1] | 1 | 13 | NA | 0[0-12.8] | 1 | 13 | NA |
| United States of America | 26[0.4-69] | 3 | 703 | 1[0.9-1] | 92.5[83.5-98.3] | 1 | 53 | NA | 58.5[44.9-71.5] | 1 | 53 | NA | -- | -- | -- | -- | 62.3[48.7-74.9] | 1 | 53 | NA |
| Saudi Arabia | 41.5[33.3-49.9] | 1 | 135 | NA | 87.5[77.4-95.1] | 1 | 56 | NA | -- | -- | -- | -- | -- | -- | -- | -- | -- | -- | -- | -- |
| Greece | 71.1[56.9-83.6] | 1 | 45 | NA | -- | -- | -- | -- | -- | -- | -- | -- | -- | -- | -- | -- | -- | -- | -- | -- |
| Finland | 10.5[8.3-13] | 10 | 6194 | 0.9[0.8-0.9] | 14.9[7.3-24.6] | 1 | 67 | NA | 5.9[0-33.7] | 2 | 112 | 0.9[0.8-1] | 5.5[0.7-13.4] | 2 | 112 | 0.5 | 8.8[2-19.1] | 2 | 112 | 0.6[0-0.9] |
| Poland | 21.1[5.2-42.7] | 1 | 19 | NA | -- | -- | -- | -- | -- | -- | -- | -- | -- | -- | -- | -- | -- | -- | -- | -- |
| South Africa | 7.3[4.4-10.8] | 1 | 260 | NA | -- | -- | -- | -- | 15.8[2.3-36.2] | 1 | 19 | NA | 26.3[8.5-48.8] | 1 | 19 | NA | 10.5[0.2-29.2] | 1 | 19 | NA |
| Israel | 30.9[23.8-38.5] | 1 | 152 | NA | -- | -- | -- | -- | -- | -- | -- | -- | -- | -- | -- | -- | -- | -- | -- | -- |
| Japan | 29.7[12.9-49.7] | 2 | 169 | 0.8[0.3-1] | -- | -- | -- | -- | 49.6[29.9-69.2] | 2 | 65 | 0.6[0-0.9] | 61.7[49.1-73.5] | 2 | 65 | 0 | 17.5[0-56.4] | 2 | 65 | 0.9[0.7-1] |
| Chile | 1.7[0-5.2] | 1 | 115 | NA | -- | -- | -- | -- | -- | -- | -- | -- | -- | -- | -- | -- | -- | -- | -- | -- |
| Australia | 27.8[21.5-34.6] | 1 | 180 | NA | -- | -- | -- | -- | 38[25-51.9] | 1 | 50 | NA | -- | -- | -- | -- | 44[30.4-58] | 1 | 50 | NA |
| Russian Federation | 0.6[0-2.4] | 1 | 179 | NA | -- | -- | -- | -- | -- | -- | -- | -- | -- | -- | -- | -- | -- | -- | -- | -- |
| **World Bank Income Groups** |  |  |  |  |  |  |  |  |  |  |  |  |  |  |  |  |  |  |  |  |
| HIC | 18.3[11.8-25.8] | 22 | 7853 | 0.9[0.9-1] | 67.4[15.4-100] | 3 | 176 | 1[1-1] | 31.4[11.3-55.8] | 6 | 280 | 0.9[0.9-1] | 29.1[2.6-67.3] | 4 | 177 | 1[0.9-1] | 24.5[7.9-46.1] | 6 | 280 | 0.9[0.9-1] |
| UMIC | 8.2[0-26.9] | 3 | 489 | 0.9[0.9-1] | -- | -- | -- | -- | 18.5[6.2-34.7] | 2 | 32 | 0 | 17.3[3-37.8] | 2 | 32 | 0.4 | 4.2[0-19.4] | 2 | 32 | 0.4 |
| **Data collection** |  |  |  |  |  |  |  |  |  |  |  |  |  |  |  |  |  |  |  |  |
| Year-round | 13.2[8.8-18.3] | 22 | 8115 | 0.9[0.9-0.9] | 55.6[0-100] | 2 | 120 | 1[1-1] | 28.5[12.8-47.2] | 8 | 312 | 0.9[0.9-1] | 24.9[5.8-50.6] | 6 | 209 | 0.9[0.9-1] | 18.9[5.8-36.5] | 8 | 312 | 0.9[0.9-0.9] |
| Seasonal | 50.7[31.5-69.8] | 3 | 227 | 0.8[0.5-0.9] | 87.5[77.4-95.1] | 1 | 56 | NA | -- | -- | -- | -- | -- | -- | -- | -- | -- | -- | -- | -- |
| **Age range** |  |  |  |  |  |  |  |  |  |  |  |  |  |  |  |  |  |  |  |  |
| 0-59 months | 9.3[4.8-15] | 10 | 2420 | 0.9[0.9-1] | -- | -- | -- | -- | 38.4[22.3-55.8] | 4 | 134 | 0.7[0.2-0.9] | 51.4[27.7-74.8] | 3 | 84 | 0.8[0.2-0.9] | 22.1[5.8-44.3] | 4 | 134 | 0.9[0.7-0.9] |
| 0-23 months | 23[13.2-34.4] | 14 | 5770 | 1[0.9-1] | 67.4[15.4-100] | 3 | 176 | 1[1-1] | 20.1[0.9-51.9] | 4 | 178 | 1[0.9-1] | 5.4[1.1-11.7] | 3 | 125 | 0.1[0-0.9] | 15.8[0-47.4] | 4 | 178 | 0.9[0.9-1] |
| 0-11 months | 30.9[23.8-38.5] | 1 | 152 | NA | -- | -- | -- | -- | -- | -- | -- | -- | -- | -- | -- | -- | -- | -- | -- | -- |
| **Clinical specimen for test** |  |  |  |  |  |  |  |  |  |  |  |  |  |  |  |  |  |  |  |  |
| MEF | 20.9[5.6-42.1] | 5 | 455 | 1[1-1] | 51.6[0-100] | 2 | 123 | 1[1-1] | 15.4[3-33.4] | 5 | 170 | 0.9[0.7-0.9] | 19.1[1.9-45.7] | 5 | 170 | 0.9[0.8-1] | 6.6[2.4-12.4] | 5 | 170 | 0.2[0-0.8] |
| MEF; NP samples | 10.9[5.8-17.4] | 8 | 4295 | 0.9[0.9-1] | -- | -- | -- | -- | 38[25-51.9] | 1 | 50 | NA | -- | -- | -- | -- | 44[30.4-58] | 1 | 50 | NA |
| NP samples | 18[5-36.6] | 6 | 1808 | 0.9[0.9-1] | 92.5[83.5-98.3] | 1 | 53 | NA | 58.7[48.4-68.7] | 2 | 92 | 0 | 56.4[40.5-71.7] | 1 | 39 | NA | 49.4[24.5-74.5] | 2 | 92 | 0.8[0.3-1] |
| Nasal samples; NP samples | 5.5[3.5-7.8] | 1 | 440 | NA | -- | -- | -- | -- | -- | -- | -- | -- | -- | -- | -- | -- | -- | -- | -- | -- |
| MEF; Nasal samples | 70.8[50.8-87.6] | 1 | 24 | NA | -- | -- | -- | -- | -- | -- | -- | -- | -- | -- | -- | -- | -- | -- | -- | -- |
| MEF; NP samples; acute and convalescent phase sera | 16.1[12.8-19.7] | 1 | 447 | NA | -- | -- | -- | -- | -- | -- | -- | -- | -- | -- | -- | -- | -- | -- | -- | -- |
| Nasal samples | 20.6[9.6-34.4] | 3 | 873 | 0.9[0.8-1] | -- | -- | -- | -- | -- | -- | -- | -- | -- | -- | -- | -- | -- | -- | -- | -- |
| **Diagnostic test** |  |  |  |  |  |  |  |  |  |  |  |  |  |  |  |  |  |  |  |  |
| Culture | -- | -- | -- | -- | 51.6[0-100] | 2 | 123 | 1[1-1] | 22.2[6.3-43.5] | 6 | 233 | 0.9[0.9-1] | 17.3[2.7-39.3] | 5 | 183 | 0.9[0.8-1] | 15.9[4.3-32] | 6 | 233 | 0.9[0.7-0.9] |
| Culture; EIA; PCR | 5.5[3.5-7.8] | 1 | 440 | NA | -- | -- | -- | -- | -- | -- | -- | -- | -- | -- | -- | -- | -- | -- | -- | -- |
| Culture; ELISA | 41.5[33.3-49.9] | 1 | 135 | NA | -- | -- | -- | -- | -- | -- | -- | -- | -- | -- | -- | -- | -- | -- | -- | -- |
| Culture; immunofluorescence assay; PCR | 7.3[4.4-10.8] | 1 | 260 | NA | -- | -- | -- | -- | -- | -- | -- | -- | -- | -- | -- | -- | -- | -- | -- | -- |
| Culture; Indirect immunofluorescence | 1.7[0-5.2] | 1 | 115 | NA | -- | -- | -- | -- | -- | -- | -- | -- | -- | -- | -- | -- | -- | -- | -- | -- |
| Culture; PCR | 25.1[20.5-30] | 3 | 352 | 0[0-0.9] | -- | -- | -- | -- | 38.5[20.5-58.1] | 1 | 26 | NA | 69.2[49.9-85.7] | 1 | 26 | NA | 3.8[0-15.8] | 1 | 26 | NA |
| Culture; TRFIA | 9.5[4.1-16.9] | 3 | 1443 | 0.9[0.9-1] | -- | -- | -- | -- | -- | -- | -- | -- | -- | -- | -- | -- | -- | -- | -- | -- |
| Direct fluorescence assay | 71.1[56.9-83.6] | 1 | 45 | NA | -- | -- | -- | -- | -- | -- | -- | -- | -- | -- | -- | -- | -- | -- | -- | -- |
| ELISA; PCR | 70.8[50.8-87.6] | 1 | 24 | NA | -- | -- | -- | -- | -- | -- | -- | -- | -- | -- | -- | -- | -- | -- | -- | -- |
| Enzyme immunoassay (IgG); TRFIA | 16.1[12.8-19.7] | 1 | 447 | NA | -- | -- | -- | -- | -- | -- | -- | -- | -- | -- | -- | -- | -- | -- | -- | -- |
| Immunochromatographic test | 21.1[5.2-42.7] | 1 | 19 | NA | -- | -- | -- | -- | -- | -- | -- | -- | -- | -- | -- | -- | -- | -- | -- | -- |
| Immunofluorescence; PCR | 9.9[5.5-15.5] | 1 | 141 | NA | -- | -- | -- | -- | -- | -- | -- | -- | -- | -- | -- | -- | -- | -- | -- | -- |
| PCR | 14.2[1.7-34.9] | 4 | 509 | 1[0.9-1] | 92.5[83.5-98.3] | 1 | 53 | NA | 58.5[44.9-71.5] | 1 | 53 | NA | -- | -- | -- | -- | 62.3[48.7-74.9] | 1 | 53 | NA |
| PCR; Antigen test | 12.8[10.4-15.4] | 1 | 702 | NA | -- | -- | -- | -- | -- | -- | -- | -- | -- | -- | -- | -- | -- | -- | -- | -- |
| TRFIA | 9.6[8.6-10.6] | 3 | 3240 | 0.1[0-0.9] | -- | -- | -- | -- | -- | -- | -- | -- | -- | -- | -- | -- | -- | -- | -- | -- |
| TRFIA; PCR | 8.8[5.9-12.2] | 1 | 318 | NA | -- | -- | -- | -- | -- | -- | -- | -- | -- | -- | -- | -- | -- | -- | -- | -- |

Bacteria 1+: at least one bacterium. "--" = No studies available for the specific category. "NA" = Not Applicable due to the presence of only one study, preventing heterogeneity analysis (I² calculation).

# Reference

Andrade, M.A., Hoberman, A., Glustein, J., Paradise, J.L., Wald, E.R., 1998. Acute otitis media in children with bronchiolitis. Pediatrics 101, 617-619.

Aymard, M., Bebear, C., Valette, M., Lina, B., Layani, M.P., De Barbeyrac, B., Orfila, J., 1997. Review on a microbiological survey and literature: Viral aetiology of cold, pharyngitis and otitis media in infants. A prospective study (winter 1995-96) results. Medecine et Maladies Infectieuses 27, 456-471.

Bulut, Y., Guven, M., Otlu, B., Yenisehirli, G., Aladag, I., Eyibilen, A., Dogru, S., 2007. Acute otitis media and respiratory viruses. European journal of pediatrics 166, 223-228.

Chonmaitree, T., Revai, K., Grady, J.J., Clos, A., Patel, J.A., Nair, S., Fan, J., Henrickson, K.J., 2008. Viral upper respiratory tract infection and otitis media complication in young children. Clinical infectious diseases : an official publication of the Infectious Diseases Society of America 46, 815-823.

Diaz, A., Bunsow, E., Mertz, S., Wang, H., Salamon, D., Leber, A., Ramilo, O., Mejias, A., 2018. Nasopharyngeal Bacterial colonization and acute otitis media in children with RSV Respiratory infection. Journal of the Pediatric Infectious Diseases Society 7, S90.

Gomaa, M.A., Galal, O., Mahmoud, M.S., 2012. Risk of acute otitis media in relation to acute bronchiolitis in children. International journal of pediatric otorhinolaryngology 76, 49-51.

Kafetzis, D.A., Astra, H., Tsolia, M., Liapi, G., Mathioudakis, J., Kallergi, K., 2003. Otitis and respiratory distress episodes following a respiratory syncytial virus infection. Clinical microbiology and infection : the official publication of the European Society of Clinical Microbiology and Infectious Diseases 9, 1006-1010.

Kleemola, M., Nokso-Koivisto, J., Herva, E., Syrjanen, R., Lahdenkari, M., Kilpi, T., Hovi, T., 2006. Is there any specific association between respiratory viruses and bacteria in acute otitis media of young children? The Journal of infection 52, 181-187.

Kotikoski, M.J., Palmu, A.A.I., Nokso-Koivisto, J., Kleemola, M., 2002. Evaluation of the role of respiratory viruses in acute myringitis in children less than two years of age. The Pediatric infectious disease journal 21, 636-641.

Kuczborska, K., Rustecka, A., Wawrzyniak, A., Bedzichowska, A., Kalicki, B., 2021. Manifestations and risk factors in children hospitalized with respiratory syncytial virus infection. Archives of Pediatric Infectious Diseases 9, 1-9.

Madhi, S.A., Govender, N., Dayal, K., Devadiga, R., Van Dyke, M.K., Van Niekerk, N., Cutland, C.L., Adrian, P.V., Nunes, M.C., 2015. Bacterial and respiratory viral interactions in the etiology of acute otitis media in HIV-infected and HIV-uninfected South African Children. Pediatr. Infect. Dis. J. 34, 753-760.

Marom, T., Israel, O., Gavriel, H., Pitaro, J., Baker, A.A., Eviatar, E., 2017. Comparison of first year of life acute otitis media admissions before and after the 13-valent pneumococcal conjugate vaccine. International journal of pediatric otorhinolaryngology 97, 251-256.

Monobe, H., Ishibashi, T., Nomura, Y., Shinogami, M., Yano, J., 2003. Role of respiratory viruses in children with acute otitis media. International journal of pediatric otorhinolaryngology 67, 801-806.

Nokso-Koivisto, J., Raty, R., Blomqvist, S., Kleemola, M., Syrjanen, R., Pitkaranta, A., Kilpi, T., Hovi, T., 2004. Presence of specific viruses in the middle ear fluids and respiratory secretions of young children with acute otitis media. Journal of medical virology 72, 241-248.

Papan, C., Willersinn, M., Weiß, C., Karremann, M., Schroten, H., Tenenbaum, T., 2020. Antibiotic utilization in hospitalized children under 2 years of age with influenza or respiratory syncytial virus infection - a comparative, retrospective analysis. BMC Infect Dis 20, 606.

Pettigrew, M.M., Gent, J.F., Pyles, R.B., Miller, A.L., Nokso-Koivisto, J., Chonmaitree, T., 2011. Viral-bacterial interactions and risk of acute otitis media complicating upper respiratory tract infection. Journal of clinical microbiology 49, 3750-3755.

Pitkaranta, A., Jero, J., Arruda, E., Virolainen, A., Hayden, F.G., 1998. Polymerase chain reaction-based detection of rhinovirus, respiratory syncytial virus, and coronavirus in otitis media with effusion. The Journal of pediatrics 133, 390-394.

Raty, R., Ziegler, T., Kleemola, M., 2004. The value of virus serology in epidemiological studies of acute otitis media in children. Journal of clinical virology : the official publication of the Pan American Society for Clinical Virology 29, 315-319.

Rosenblüt, A., Santolaya, M.E., González, P., Corbalán, V., Avendaño, L.F., Martínez, M.A., Hormazabal, J.C., 2001. Bacterial and viral etiology of acute otitis media in Chilean children. PEDIATRIC INFECTIOUS DISEASE JOURNAL 20, 501-507.

Ruohola, A., Pettigrew, M.M., Lindholm, L., Jalava, J., Raisanen, K.S., Vainionpaa, R., Waris, M., Tahtinen, P.A., Laine, M.K., Lahti, E., Ruuskanen, O., Huovinen, P., 2013. Bacterial and viral interactions within the nasopharynx contribute to the risk of acute otitis media. The Journal of infection 66, 247-254.

Sawada, S., Okutani, F., Kobayashi, T., 2019. Comprehensive Detection of Respiratory Bacterial and Viral Pathogens in the Middle Ear Fluid and Nasopharynx of Pediatric Patients With Acute Otitis Media. The Pediatric infectious disease journal 38, 1199-1203.

Toivonen, L., Karppinen, S., Schuez-Havupalo, L., Teros-Jaakkola, T., Mertsola, J., Waris, M., Peltola, V., 2020. Respiratory syncytial virus infections in children 0-24 months of age in the community. Journal of Infection 80, 69-75.

Tomochika, K., Ichiyama, T., Shimogori, H., Sugahara, K., Yamashita, H., Furukawa, S., 2009. Clinical characteristics of respiratory syncytial virus infection-associated acute otitis media. Pediatrics international : official journal of the Japan Pediatric Society 51, 484-487.

Uitti, J.M., Tahtinen, P.A., Laine, M.K., Huovinen, P., Ruuskanen, O., Ruohola, A., 2015. Role of Nasopharyngeal Bacteria and Respiratory Viruses in Acute Symptoms of Young Children. The Pediatric infectious disease journal 34, 1056-1062.

Vesa, S., Kleemola, M., Blomqvist, S., Takala, A., Kilpi, T., Hovi, T., 2001. Epidemiology of documented viral respiratory infections and acute otitis media in a cohort of children followed from two to twenty-four months of age. The Pediatric infectious disease journal 20, 574-581.

Wiertsema, S.P., Chidlow, G.R., Kirkham, L.-A.S., Corscadden, K.J., Mowe, E.N., Vijayasekaran, S., Coates, H.L., Harnett, G.B., Richmond, P.C., 2011. High detection rates of nucleic acids of a wide range of respiratory viruses in the nasopharynx and the middle ear of children with a history of recurrent acute otitis media. Journal of medical virology 83, 2008-2017.

Yatsyshina, S., Mayanskiy, N., Shipulina, O., Kulichenko, T., Alyabieva, N., Katosova, L., Lazareva, A., Skachkova, T., Elkina, M., Matosova, S., Shipulin, G., 2016. Detection of respiratory pathogens in pediatric acute otitis media by PCR and comparison of findings in the middle ear and nasopharynx. Diagn. Microbiol. Infect. Dis. 85, 125-130.
